# Carbon quantum dots in plant photosynthesis: from material design to agricultural performance

**DOI:** 10.1039/d6ra07036a

**Published:** 2026-09-03

**Authors:** Rishabh, Moushumi Ghosh

**Affiliations:** a TIET-TAU Center of Excellence for Food Security, Thapar Institute of Engineering and Technology Patiala Punjab-147004 India; b Department of Biotechnology, Thapar Institute of Engineering and Technology Patiala Punjab-147004 India mghosh@thapar.edu

## Abstract

Sustainable agricultural intensification requires nanomaterials that regulate plant physiology while improving resource-use efficiency and stress resilience. Carbon quantum dots (CQDs) have emerged as photosynthetic nano-regulators because their optical and electronic properties, surface functionality, dispersibility, and compositional flexibility enable control over plant uptake and activity. This review develops a structure-function-performance framework linking CQD physicochemical characteristics, including particle size, surface chemistry, optical properties, and heteroatom doping, with transport and photosynthetic regulation. Five interconnected mechanisms are identified: enhanced electron transport, improved light harvesting and spectral conversion, redox homeostasis and antioxidant regulation, chlorophyll biosynthesis and protection, and enhanced carbon fixation. Their evidentiary strength varies, with chloroplast-level evidence providing the strongest support for CQD-mediated electron-transport enhancement, whereas the other mechanisms are supported primarily by physiological, biochemical, photochemical, molecular, and transcriptomic observations. Multi-omics studies further indicate that CQD responses extend beyond photosynthesis into nutrient acquisition, carbon and nitrogen metabolism, and stress adaptation. Reported applications include seed priming, photosynthetic enhancement, abiotic-stress mitigation, nutrient-use efficiency, and spectral-conversion strategies. However, limited long-term field validation, and uncertainties surrounding scalability, economic viability, environmental fate, and soil-climate dependence remain major barriers to agricultural translation. This review identifies mechanistic gaps and priorities for developing reproducible, environmentally responsible CQD technologies.

## Abstract 2

Sustainable agriculture requires nature-inspired nanomaterials that precisely regulate plant physiology, enhance photosynthesis, improve resource-use efficiency, and strengthen resilience to environmental stress.

## Graphical abstract

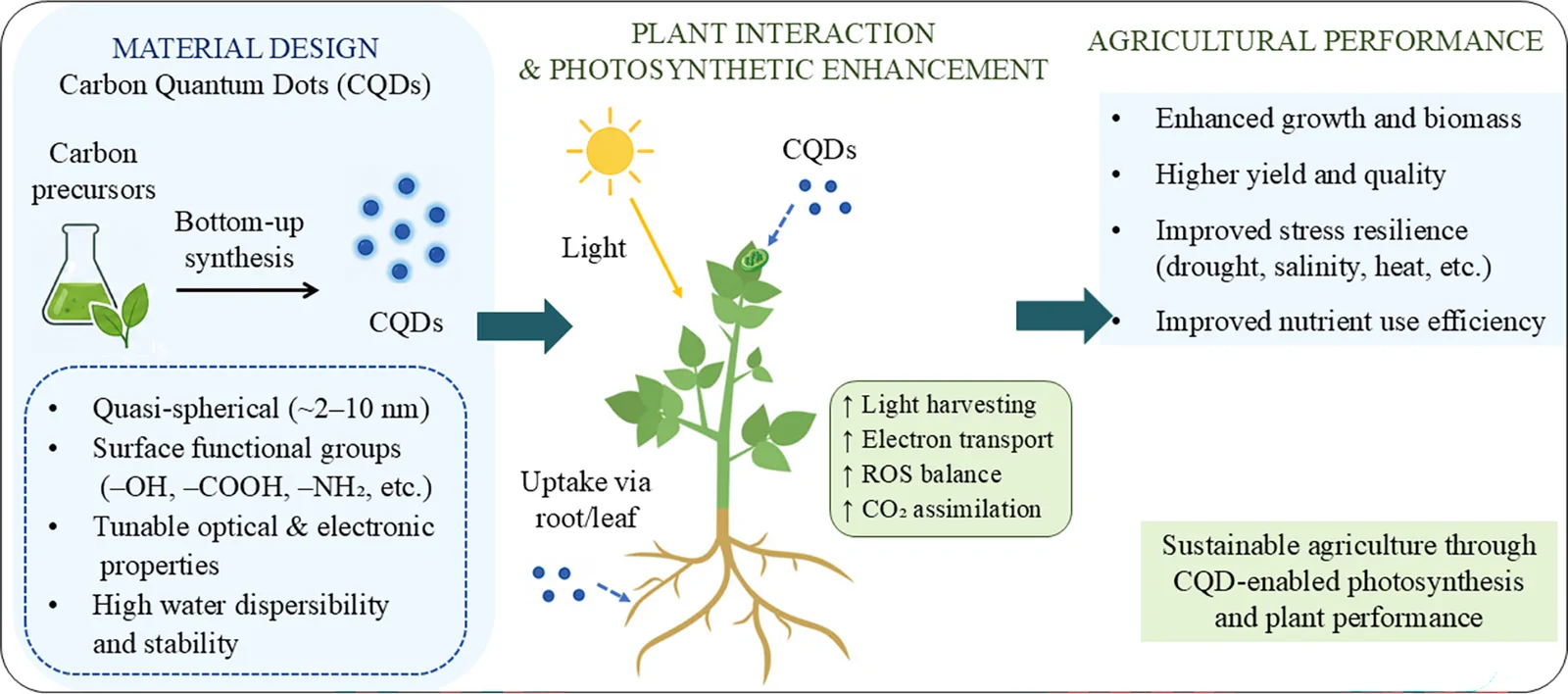

**Environmental significance**

Improving crop productivity while reducing environmental inputs is a major challenge for sustainable agriculture. Carbon quantum dots (CQDs) have emerged as promising nanomaterials due to their tunable physicochemical properties, biocompatibility, and compatibility with green synthesis approaches. This review critically evaluates CQDs as photosynthetic nano-regulators, emphasizing how rational design strategies influence plant uptake, photosynthetic performance, stress resilience, nutrient-use efficiency, and crop productivity. By integrating current knowledge on structure-function-performance relationships, the review highlights the potential of CQDs to enhance agricultural sustainability through improved resource-use efficiency, reduced dependence on agrochemicals, and greater climate resilience. It also identifies key challenges related to mechanistic understanding, biosafety, environmental fate, and field-scale implementation. The insights presented provide a framework for developing environmentally responsible nanotechnologies that support sustainable and climate-resilient food production systems.

## 1 Introduction

Feeding a growing population without further degrading agriculture's resource base is among the defining challenges of this century. Climate change, water scarcity, soil degradation, nutrient depletion, salinization, heavy metal contamination, and emerging biotic stresses are converging to constrain yields as demand continues to rise.^1–6^ Assessments of crop physiology under warming and water-limited conditions,^7^ together with systems-level food security analyses,^8^ reach a similar conclusion: incremental adjustments to existing practices are unlikely to be sufficient. The intensive fertilizer and pesticide regimes that drove twentieth-century yield gains have reached diminishing returns, and their environmental costs, including biodiversity loss, declining resource-use efficiency, and persistent contamination, are now widely recognized as unsustainable.^5,9,10^ Nanotechnology has consequently attracted sustained interest as a route to higher productivity and stress resilience with a smaller environmental footprint.^11–14,142^ Reviews of the field describe a shift from nanomaterials regarded as a laboratory curiosity to a genuine input category spanning fertilization, crop protection, water management, and diagnostics.^15–17^ Nanofertilizers illustrate this logic well: encapsulating or surface-loading nutrients at the nanoscale can slow release, reduce leaching, and raise nutrient-use efficiency relative to conventional formulations,^18^ although economic analyses caution that these efficiency gains must be weighed against production cost and formulation complexity before such materials can be adopted at scale.^19^ Particle size, surface charge, and coating chemistry govern whether a nanomaterial is absorbed at the root or leaf, how far it travels, and whether it accumulates in edible tissue,^20–23^ and toxicological screening has therefore become a standard companion to efficacy testing.^24^

Carbon-based nanomaterials occupy a distinctive position within this broader effort. Carbon dots (CDs) were identified, somewhat by accident, during the electrophoretic purification of single-walled carbon nanotube fragments,^25^ and the discovery that small, surface-passivated carbon nanoparticles could fluoresce brightly and in tunable colours established them as a class in their own right rather than a nanotube by-product.^26^ The carbon dot family has since been organized into several related subtypes, including graphene quantum dots, carbon nanodots, and carbonized polymer dots, distinguished by core crystallinity and surface chemistry rather than by a single unifying structure.^27^ Graphene quantum dots, in particular, have developed into a substantial sub-literature of their own, valued for their bandgap tunability and edge-state luminescence.^28,29^ Carbon quantum dots (CQDs) are attractive for agricultural use because of the same property set that first drove their adoption elsewhere: an ultrasmall, quasi-spherical structure typically under 10 nm, tunable optical and electronic behaviour, dense surface functionality, strong aqueous dispersibility, and high biocompatibility.^30–33^ These properties have already supported established application areas in sensing, bioimaging, catalysis, environmental remediation, and energy conversion,^34–37^ with dedicated literature addressing CQD-based biosensing and therapeutics,^38^ green chemistry approaches to synthesis and sensing,^39^ photocatalytic and electrocatalytic dopant engineering,^40,41^ and energy storage composites.^42^ The original biocompatible carbon nanodot ink reported by Qu *et al.*^132^ remains a frequently cited early proof of concept for biological applications. Heteroatom doping has been a key strategy underlying this versatility. The incorporation of nitrogen, sulfur, or other heteroatoms during synthesis modulates the electronic structure and surface chemistry of CQDs, thereby tailoring their properties for specific applications.^149^ This approach recurs as a theme across nearly all application areas of CQDs.

These same properties translate readily to agriculture. Green synthesis routes now convert biomass, agricultural residues, food-processing waste, algae, and medicinal plants directly into CQDs, aligning nanomaterial production with circular bioeconomy goals rather than competing with them.^43–45^ Reviews specifically addressing CDs in agricultural and plant systems have grown in parallel, surveying effects on germination, growth, and stress tolerance across crop species.^46–48^ Particle size, surface charge, functional group identity, and heteroatom composition jointly determine tissue distribution, transport route, and biological activity once CQDs enter plant tissue,^49–53^ a pattern consistent with the size and coating-dependent uptake behavior documented for other nanomaterials applied to leaves.^54^ Photosynthetic regulation has become the most actively pursued of the functions that follow from this transport capacity. The conceptual foundation was laid by plant nanobionics, in which engineered nanomaterials were shown to interface directly with chloroplasts and enhance photosynthetic activity rather than simply supplying nutrients.^55,56^ CQDs extend this logic through optical, electronic, and redox properties that allow them to engage the photosynthetic apparatus directly, and the same fluorescence that makes CQDs useful as biosensors elsewhere has begun to support real-time monitoring of plant physiological state, paralleling a broader move toward wearable and nanoscale sensors for precision agriculture.^57–59^ Findings of this kind motivate a conceptual shift: CQDs are better understood as photosynthetic nano-regulators than as generic plant growth stimulants, a shift this review develops fully in the sections that follow.

This term is used deliberately throughout the review and warrants an explicit definition before the argument proceeds. Here, a photosynthetic nano-regulator is defined as an engineered nanomaterial that alters photosynthetic rate or efficiency through direct physicochemical interaction with the photosynthetic apparatus, acting on light harvesting, the photosynthetic electron transport chain, adenosine triphosphate (ATP) and nicotinamide adenine dinucleotide phosphate (NADPH) generation, carbon fixation, or the redox and signaling networks that protect this machinery, rather than acting only by supplying a limiting nutrient. This is a functional definition rather than a compositional one: what qualifies a material is its mode of action, not its size class or chemical identity. The definition draws an explicit distinction from conventional plant growth-promoting nanomaterials, including nanofertilizers and most nutrient-delivery nanocarriers, which act indirectly. Those materials encapsulate, stabilize, or slow-release an already essential nutrient, and any resulting improvement in photosynthesis is a downstream consequence of relieving nutrient limitation rather than of the nanomaterial engaging the photosynthetic apparatus directly.^18^ CQDs can and frequently do also improve nutrient status, but this route is supplementary rather than sufficient to explain their effects, a claim the mechanistic evidence assembled in Section 5 directly supports. The distinction is not merely semantic. It determines which physicochemical properties matter for rational design, namely electronic structure and redox activity rather than nutrient loading capacity alone, and which biological readouts are appropriate for evaluating a candidate material: electron transport rate, (Photosystem II) PSII efficiency, and ribulose-1,5-bisphosphate carboxylase/oxygenase (Rubisco) activity, considered alongside rather than in place of tissue nutrient content.

These advances, however, have outpaced the field's conceptual integration. Evidence for CQD-mediated photosynthetic enhancement is distributed across materials science, plant physiology, and agricultural engineering, and is rarely assembled into a single account of which structural features drive which biological outcome, or how strongly each proposed mechanism is actually supported. Section 2 examines this gap in relation to the existing review literature; the remainder of this article then develops a structure-function-performance framework that connects CQD design directly to photosynthetic regulation and agronomic outcome.

## 2 Need for the review

The CQD-agriculture literature has grown quickly, propelled by advances in green synthesis, heteroatom engineering, and multifunctional design. CQDs have moved from a niche sensing and bioimaging material into a platform credited with enhancing photosynthesis, nutrient-use efficiency, and stress tolerance across a growing list of crops. This growth has already produced six substantive reviews spanning synthesis, physicochemical properties, plant interactions, toxicity, soil applications, and agricultural performance.^47,60–64^Table 1 situates the present review against each of these. Read together, these reviews share a structural limitation. Most treat material development and biological response as separate literature instead of as two halves of a single causal chain. Synthesis, plant growth promotion, stress mitigation, and field application are typically discussed in sequence rather than in a mechanistic framework. The relationships that matter most for rational design, including how precursor choice, particle size, surface chemistry, heteroatom doping, and optical and electronic structure translate into uptake, photosynthetic regulation, and agronomic outcomes, remain underspecified.

**Table 1:** Comparative analysis of existing reviews and the distinct scope of the present review

S. No.	Review title	Journal	Main focus	Key contributions	Remaining gaps	Contribution of the present review	References
1	An Insight into the role of carbon dots in the agriculture system	*Environmental Research*	CDs in agriculture	Established CDs as multifunctional materials for growth promotion, photosynthesis enhancement, stress mitigation, and sensing	Lacks structure-function analysis	Links CQD design parameters with plant physiological responses	47
2	Carbon quantum dots as versatile nanomaterials for improving soil health and plant stress tolerance	*Journal of Agricultural and Food Chemistry*	CQD synthesis, soil health, and stress tolerance	Comprehensive overview of synthesis, soil interactions, and toxicity	No unified design-performance framework	Connects CQD structure, transport, photosynthesis, and agricultural outcomes	60
3	Carbon dots in agriculture: fundamentals, applications and perspectives	*Coordination Chemistry Reviews*	Fundamentals and agricultural applications	Detailed discussion of synthesis, photoluminescence, and applications	Weak linkage between material properties and plant responses	Establishes structure-function-performance relationships	61
4	Carbon dots in green agriculture: Next-Generation Tool for photosynthesis enhancement and agricultural resilience	*Chemical Engineering Journal*	Photosynthesis enhancement and resilience	Discussed uptake pathways and biological functions	Limited analysis of governing material properties	Identifies physicochemical determinants of photosynthetic regulation	62
5	Mechanistic insights into carbon nanomaterials as potential plant biostimulants	*Plant Physiology and Biochemistry*	Carbon nanomaterials as plant biostimulants	Examined uptake, photosynthesis, stress responses, and omics data	CQD-specific relationships remain unclear	Develops a CQD-centered mechanistic framework	63
6	Biomassderived carbon dots for agriculture: ROS-mediated stress tolerance, postharvest preservation, and designing for safety	*Trends in Food Science & Technology*	Biomass-derived CDs and ROS regulation	Introduced ROS-mediated and safe-by-design concepts	Focused mainly on ROS biology and postharvest applications	Expands to photosynthetic engineering, carbon fixation, nutrient acquisition, and multifunctional CQD design	64
7	Carbon quantum dots in plant photosynthesis: from material design to agricultural performance	This review	Integrates CQD design, structural engineering, uptake, transport, chloroplast localization, photosynthetic regulation, seed priming, stress resilience, nanozyme activity, and agricultural applications	Addresses the lack of a unified design-to-performance framework	—	Establishes a comprehensive structure-function-performance paradigm linking CQD engineering with photosynthetic regulation, crop productivity, and sustainable agriculture	This review

A second, more consequential gap is the absence of a framework for weighing mechanistic evidence on its own terms. Reports of higher chlorophyll content, photosynthetic efficiency, biomass, and stress resilience following CQD treatment are now common, but the studies supporting them differ markedly in mechanistic rigor. Direct, chloroplast-level evidence supports CQD-facilitated electron transport and energy conversion.^65,66^ Spectral conversion, artificial antenna effects, chloroplast targeting, reactive oxygen species (ROS)-mediated photoprotection, and metabolic reprogramming are primarily supported by physiological, biochemical, transcriptomic, or metabolomic correlations.^67–71^ Without an explicit hierarchy of evidence, these mechanisms risk being treated as equally established when they are not. Recent advances in engineering and omics further sharpen this need. Heteroatom-doped, co-doped, spectral-converting, nanozyme-like, and stress-responsive CQDs have substantially widened the functional landscape of agricultural nanotechnology.^72–75^ Multi-omics studies show that CQDs can regulate physiological networks spanning photosynthesis, carbon metabolism, nutrient acquisition, and environmental adaptation.^68,71,76^ Neither development has yet been folded into a coherent structure-function-performance account. Translational questions compound these mechanistic ones. Uptake pathways, intracellular localization, long-term environmental fate, biosafety, the scalability of green synthesis, and field performance all remain open questions, each representing a real obstacle between laboratory promise and agricultural deployment.

This review addresses these gaps by adopting a structure-function-performance perspective that links CQD design, plant transport, chloroplast-associated interaction, photosynthetic regulation, and agricultural outcome within a single account. It departs from prior reviews (Table 1) on three points. It grades the evidence supporting each proposed photosynthetic mechanism rather than reporting mechanisms at face value. It treats materials engineering and plant physiology as a single causal chain rather than as parallel literature. And it uses that chain to identify where rational CQD design is already possible and where it remains aspirational. The aim is to develop a framework specific enough to guide the next generation of photosynthetic nano-regulators for climate-resilient, resource-efficient agriculture.

## 3 Structural engineering strategies of CQDs for agricultural applications

Five physicochemical levers largely determine how a CQD performs once it enters a plant: particle size, surface chemistry, electronic structure, charge distribution, and optical behavior. Each can be tuned through precursor selection, synthesis methodology, heteroatom incorporation, and post-synthetic functionalization. This is what makes rational design possible at all. A CQD optimized for uptake, photosynthetic regulation, nutrient acquisition, or stress resilience is, in practice, a CQD whose synthesis has been deliberately matched to that goal. The sections below work through these levers in turn. Table 2 summarizes the resulting engineering strategies alongside their functional outcomes.

**Table 2:** Structure-function relationships governing CQDs design for photosynthetic nano-regulator and agricultural applications

S. No.	Engineering parameter	Design strategy	Effect on CQD properties	Mechanistic basis	Observed biological function	Agricultural relevance	Limitation	References
1	Precursor engineering	Small-molecule and biomass-derived precursors (citric acid, glucose, citrus peels, rice residues, algae, medicinal plants)	Tunable size, surface chemistry, and heteroatom content	Controls defect density, surface states, and functional groups	Improved biocompatibility and plant interactions	Sustainable agricultural nanomaterials	Batch-to-batch variability in biomass composition limits reproducibility and standardization	30, 33, 43–45 and 64
2	Synthesis engineering	Hydrothermal, microwave, solvothermal synthesis	Tunable graphitization, dispersibility, and optical properties	Controls electronic structure and photophysical behavior	Enhanced biological activity and photosynthetic performance	Scalable CQD production platforms	High energy input and equipment cost constrain large-scale, field-level production	32, 47, 61, 78 and 81
3	Size engineering	Nanoscale CQDs (2–10 nm)	Increased surface area and mobility	Facilitates penetration through plant barriers and intracellular trafficking	Enhanced uptake, transport, and tissue distribution	Efficient delivery into plant systems	Precise size control is difficult to maintain during scale-up, and narrow size ranges are needed for consistent uptake	49–51 and 53
4	Surface functionalization	Hydroxyl-, carboxyl-, and amino-rich surfaces	Improved hydrophilicity, dispersibility, charge control, and membrane affinity	Regulates interactions with seeds, roots, and cellular membranes	Enhanced germination, nutrient uptake, and cellular internalization	Improved seed priming and plant delivery	Functional groups can degrade or be lost under field conditions, reducing long-term stability	32, 53 and 79
5	Surface-charge engineering	Positively and negatively charged CQDs	Altered transport behavior and colloidal stability	Regulates apoplastic and symplastic transport pathways	Improved root-to-shoot translocation	Precision plant-delivery systems	Charge behavior is soil- and species-dependent, limiting generalizability across crop systems	52 and 53
6	Nitrogen doping	Urea, EDA, amino acids	Increased electron density, conductivity, and quantum yield	Enhanced photosynthetic electron transfer and charge mobility	Improved photosynthesis, carbon fixation, and drought tolerance	Yield enhancement and photosynthetic regulation	Dose-dependent effects are poorly characterized, with risk of adverse impact at higher concentrations	66, 67 and 87
7	Sulfur/phosphorus/boron doping	Thiourea, l-cysteine, phosphate salts, boric acid	Modified electronic structure, charge distribution, and redox activity	Improved charge separation and ROS regulation	Enhanced stress tolerance and photochemical performance	Salinity and oxidative-stress mitigation	Mechanistic understanding of dopant-specific redox pathways remains largely correlative rather than causal	33, 58, 81 and 89
8	Magnesium doping	Mg incorporation and Mg/N co-doping	Improved light harvesting and chloroplast compatibility	Chlorophyll stabilization, photoprotection, and enhanced electron transport	Increased photosynthetic efficiency and stress resilience	Salinity tolerance and UV-B protection	Evidence is largely limited to short-term greenhouse studies, with little validation under field conditions	73 and 74
9	Selenium doping	Se-containing precursors	Enhanced antioxidant and signaling activity	Activation of stress-responsive pathways and ROS regulation	Improved germination, growth, and stress adaptation	Drought and salinity resilience	Selenium toxicity thresholds in plants and soil ecosystems are not well established for CQD-bound forms	58 and 72
10	Cerium/iron doping	Ce and Fe incorporation	Enhanced electron-transfer capability and nanozyme activity	Promotion of chlorophyll biosynthesis and ROS detoxification	Improved photosynthetic protection and oxidative-stress mitigation	Heavy-metal and abiotic-stress tolerance	Long-term accumulation of metal dopants in soil and edible tissue has not been adequately assessed	75 and 92
11	Co-doping engineering	N,S-, N,P-, Ce,N-, and Mg,N-CQDs	Synergistic modulation of electronic structure and redox behavior	Enhanced charge transfer and stress regulation	Improved photosynthesis and stress adaptation	Multifunctional agricultural CQDs	Synthesis complexity increases with each additional dopant, raising cost and reducing reproducibility	67, 74 and 93
12	Optical engineering	High-quantum-yield and wavelength-converting CQDs	Enhanced light absorption and emission	UV-to-visible conversion and improved solar-energy utilization	Increased photosynthetic activity and biomass accumulation	Photosynthetic nano-regulator	Quantum yield often degrades under prolonged UV or field light exposure, limiting durability	55, 67 and 69
13	Electron-transfer engineering	Graphitic and defect-rich CQDs	Improved conductivity and charge mobility	Electron donation to photosynthetic systems and facilitation of charge separation	Increased ATP/NADPH generation and carbon fixation	Enhanced photosynthetic efficiency	Direct evidence of electron transfer into the photosynthetic chain *in planta* is still limited and largely inferential	62, 65, 66 and 70
14	Advanced functional engineering	Polymer functionalization, hydrogels, biomolecule conjugation, nanozyme engineering	Controlled release, enhanced stability, and catalytic activity	Sustained delivery, signaling regulation, and ROS scavenging	Improved seedling establishment and stress tolerance	Next-generation smart agricultural nanomaterials	Added functional layers increase formulation complexity and cost, complicating regulatory approval	75, 128 and 129
15	Precision agricultural integration	CQDs integrated with sensing and smart agriculture platforms	Real-time monitoring and targeted delivery	Coupling nanomaterials with precision farming technologies	Improved resource-use efficiency and crop management	Smart agriculture and digital farming	Integration with existing farm infrastructure and data systems remains largely untested at field scale	130 and 131
16	Biosafety and sustainable design	Green synthesis and safe-by-design CQDs	Reduced environmental footprint and toxicity	Sustainable nanomaterial development and environmental compatibility	Enhanced agricultural acceptance and field applicability	Scalable sustainable agriculture	Standardized regulatory and toxicological frameworks for CQDs in agriculture are still lacking	61, 64 and 72

### 3.1 Fundamentals and structural characteristics of CQDs

At the structural level, CQDs are quasi-spherical, sub-10-nm carbon nanomaterials whose tunable photoluminescence, dense surface functionality, and high biocompatibility were introduced in Section 1.^30–32,139^ What matters for agricultural design is the architecture beneath these properties. A carbon-rich core of graphitic, amorphous, or mixed sp^2^/sp^3^ domains is wrapped in a surface shell rich in hydroxyl, carboxyl, carbonyl, amino, and other oxygen- or nitrogen-containing groups.^30,33,77^ The core governs charge mobility, electron-transfer behavior, and optical absorption. The surface shell governs photoluminescence, colloidal stability, chemical reactivity, and biological interaction. Particle size, degree of graphitization, and surface-defect density were shown early on to jointly control this optical and electronic behavior, and that core insight still underlies most modern CQD design strategies.^78–80^

Several of these features are specifically advantageous for plant systems. Ultrasmall dimensions let CQDs cross biological barriers and move within plant tissue. Abundant surface functional groups confer aqueous stability and allow CQDs to interact directly with cellular membranes, proteins, and metabolites.^49,51,52^ Their fluorescence, electron-transfer capacity, and redox activity go a step further, enabling direct engagement with photosynthetic and metabolic machinery. This sets CQDs apart from nutrient-based agricultural nanomaterials, which act on plant performance only indirectly.^29,55,67^

This multifunctionality arises from how structural features combine, not from any single property in isolation. Particle size sets mobility and tissue distribution. Surface chemistry sets accessibility and the nature of intracellular interaction. Heteroatom incorporation resets electronic structure, charge-transfer behavior, and antioxidant activity.^33,81^ Together, these parameters determine whether a given CQD can meaningfully influence light harvesting, photosynthetic electron transport, carbon assimilation, ROS homeostasis, or stress-responsive signaling.^62,67,71,76^ Green synthesis adds a further practical dimension. CQDs derived from agricultural residues, fruit waste, algae, medicinal plants, and other renewable biomass often already include intrinsic heteroatom functionality, improved biocompatibility, and a smaller environmental footprint.^43,44,82^ These properties align naturally with sustainable agriculture and circular bioeconomy objectives, rather than requiring separate optimization. The physicochemical profile of a CQD is therefore not incidental to its biological behavior. It is the mechanism through which that behavior arises. The sections that follow examine each design lever in turn, covering precursor and synthesis choices, size and surface engineering, heteroatom doping, and integrated design principles, before turning, in Section 4, to how these engineered particles actually move through and act within the plant.

### 3.2 Precursor and synthesis engineering

Precursor composition and synthesis methodology set the physicochemical starting point from which all downstream properties follow. The carbon source, intrinsic heteroatom content, and fabrication conditions collectively determine particle size, degree of graphitization, surface functionality, defect density, and optical and electronic structure.^30,33,43^ Precursor and synthesis choice is therefore the first, and in many respects the most consequential, point of control over a CQD's eventual agricultural behavior. Small organic molecules such as citric acid, glucose, sucrose, and amino acids dominated early synthesis work because they are readily available and yield CQDs with relatively uniform physicochemical properties.^30,33^ They remain useful for mechanistic work because their well-defined structures isolate size effects, surface-state formation, charge-transfer behavior, and photoluminescence mechanisms for systematic study. Agricultural and sustainability priorities, however, have pushed synthesis toward biomass-derived precursors: agricultural residues, fruit peels, medicinal plants, algae, food-processing waste, pollen, and other renewable carbon sources now dominate the applied literature.^43–45^

Biomass-derived CQDs offer significant advantages for agricultural applications. Natural biomolecules, including carbohydrates, proteins, amino acids, polyphenols, and organic acids, provide oxygen-, nitrogen-, sulfur-, and phosphorus-containing functionalities as intrinsic dopants during carbonization. This reduces or eliminates the need for separate post-synthetic doping.^44,82^ The resulting particles typically exhibit strong aqueous dispersibility, good biocompatibility, abundant surface-active sites, and favorable environmental compatibility: precisely the properties that large-scale, low-toxicity, environmentally sustainable agricultural deployment requires. Precursor selection visibly shapes agricultural outcomes. Basil-derived CQDs improved seed-priming efficiency and early plant establishment.^83^ Algae-derived CQDs served as spectral-conversion materials, increasing photosynthetic efficiency and biomass by improving light utilization.^69^ Coffee-ground-derived CDs functioned as artificial photosynthetic antennae through excitation-energy transfer.^70^ Lawn-waste-derived CQDs improved salinity tolerance through coordinated regulation of photosynthesis and antioxidant response.^84^ The pattern across these studies is consistent: precursor chemistry is a determinant of biological function in its own right, not a synthetic detail.

Synthesis methodology then refines what precursor chemistry makes possible. Hydrothermal carbonization remains the dominant approach, valued for its simplicity, scalability, and tendency to yield highly dispersible, oxygen-rich CQDs well suited for biological applications.^47,61^ Its mild conditions allow carbonization, surface passivation, and heteroatom incorporation to proceed simultaneously. Microwave-assisted synthesis offers a faster, more energy-efficient alternative with tighter control over particle size and surface-state formation.^32,81^ Solvothermal, pyrolytic, and ultrasonic-assisted methods provide additional control over the degree of graphitization, defect density, fluorescence behavior, and electronic properties, as specific applications demand. Precursor and synthesis engineering set the optical, electronic, and redox ceiling that subsequent strategies work within. Surface modification, heteroatom doping, and multifunctional design can refine that starting material, but cannot exceed it. The following sections examine how size, surface chemistry, and doping convert that starting material into a CQD with predictable agricultural behavior.

### 3.3 Size, surface chemistry, and charge engineering

Particle size, surface chemistry, and surface charge are the three physicochemical parameters that most directly govern CQD biological performance. Together, they determine colloidal stability, plant uptake efficiency, intracellular transport, organelle accessibility, and the nature of any downstream physiological interaction.^51–53^ Particle size governs mobility first. CQDs in the typical 2–10 nm range can penetrate biological barriers, diffuse through cell wall pores, and move systemically through vascular tissue.^49,50^ Smaller particles generally reach metabolically active regions, such as meristems, vascular tissue, and chloroplast-rich cells, more efficiently than larger ones.^51^ Size cuts both ways. Particles small enough to move freely may pass through target tissue too quickly to act on it, while larger particles may simply fail to cross the relevant barrier. Biological performance in practice depends on balancing transport efficiency with tissue retention, rather than maximizing either alone.^53^

Surface chemistry is the second lever. Hydroxyl, carboxyl, carbonyl, amino, and related oxygen- or nitrogen-containing groups set aqueous dispersibility, colloidal stability, and chemical reactivity, while also shaping which biological molecules a CQD actually encounters.^32^ Hydroxyl- and carboxyl-rich surfaces tend to favor water compatibility and interaction with seed coats, root tissue, and nutrient ions. Amino-functionalized surfaces, in contrast, favor membrane affinity, which promotes internalization and intracellular transport.^49,79^ These same functional groups also influence electron-transfer capacity and ROS-scavenging activity, so surface chemistry shapes what a CQD does once it arrives, not only where it goes. Surface charge adds a third control axis. Plant cell walls and plasma membranes carry a net negative charge. Positively charged CQDs tend to interact more strongly with membrane surfaces and achieve greater cellular internalization, while negatively charged CQDs tend instead toward superior colloidal stability and aqueous mobility.^52,53^ This charge dependence carries through to root absorption, vascular translocation, tissue distribution, and ultimately biological activity itself.

None of these three parameters operates in isolation. A CQD's effective performance depends on its size, surface functionality, and charge. Nanoscale particles with hydrophilic surface groups and well-matched charge characteristically show broader tissue distribution and more reliable transport than particles optimized on only one axis. This combined optimization matters most for applications that require chloroplast accessibility, systemic stress protection, or long-distance transport. The agricultural literature supports this picture, if mostly indirectly. CQDs engineered for favorable size, surface chemistry, and charge have improved germination, nutrient acquisition, photosynthetic efficiency, biomass accumulation, and abiotic-stress tolerance across diverse crop systems.^85,86^ The improved tissue distribution observed after seed priming, root exposure, and foliar application reinforces the same conclusion.^69,76^ Size, surface chemistry, and charge define the accessibility ceiling for everything discussed in the rest of this review. A CQD cannot regulate photosynthesis, nutrient acquisition, or stress response in tissue it never reaches. Section 3.4 turns to heteroatom doping, the strategy through which CQDs are given not just access to plant tissue but a specific biological function once they arrive.

### 3.4 Heteroatom doping and functional engineering

Heteroatom doping is the most powerful single lever for tailoring the optical, electronic, and biological behavior of CQDs. Introducing nitrogen, sulfur, phosphorus, magnesium, selenium, iron, cerium, or titanium reshapes electronic structure, defect density, charge distribution, photoluminescence, and redox activity.^33,81^ This enables CQDs to be matched to specific agricultural functions, including photosynthetic enhancement, antioxidant defense, nutrient utilization, and stress adaptation, depending on the chosen element.

Nitrogen is the most thoroughly studied dopant and, on current evidence, the most reliably effective for photosynthetic enhancement. By raising electron density, conductivity, and charge mobility, nitrogen doping consistently improves photosynthetic electron transport and carbon assimilation.^66,67,87^ N-CQDs have repeatedly delivered higher photosynthetic efficiency, biomass, nutrient-use efficiency, and drought tolerance across crop systems.^62,67,88^ Phosphorus doping proceeds *via* a related mechanism, facilitating charge separation and electron transfer to support photochemical activity and energy conversion.^58,81^ Together, these modifications strengthen the interface between a CQD and the photosynthetic machinery it is meant to engage. A second function has emerged from sulfur- and selenium-doping: photoprotection against oxidative stress. Environmental stress typically damages photosynthesis by triggering excess ROS production, which degrades membranes and chlorophyll and disrupts electron transport. Sulfur-containing CQDs counteract this by enhancing antioxidant and ROS-scavenging activity.^89,145^ Selenium-doped CQDs activate antioxidant defense pathways and stress-responsive signaling, helping preserve photosynthetic machinery under salinity and drought.^72,90,91^ These findings position redox-active dopants as protective agents, not only enhancing ones, a distinction that matters for matching dopant choice to the stress a given crop system actually faces.

Magnesium doping takes a more biomimetic route. Because magnesium is intrinsic to the chlorophyll molecule itself, Mg-containing CQDs directly improve chloroplast compatibility, light harvesting, and photosystem stability, with reported gains in photosynthetic efficiency and tolerance to salinity and UV-B stress.^73,74^ Cerium doping exerts a parallel effect *via* a distinct mechanism, promoting chlorophyll biosynthesis and photochemical performance *via* redox-mediated regulation of cellular metabolism.^92^

Iron doping introduces a qualitatively different strategy. It protects catalytically rather than stoichiometrically. Fe-doped CQDs exhibit enzyme-mimetic, nanozyme-like activity that continuously scavenges ROS rather than being consumed in a single detoxification event, thereby extending their protective capacity well beyond what conventional antioxidants can sustain under prolonged stress.^75^ Titanium doping extends heteroatom engineering into molecular territory. Ti-doped CQDs improved salinity tolerance in rice through coordinated transcriptomic and metabolomic reprogramming, demonstrating that an engineered CQD can act on entire physiological networks rather than just a single biochemical step.^76^ Other omics studies echo this pattern, linking CQD-enhanced photosynthesis to broader shifts in carbon metabolism, nutrient acquisition, antioxidant defense, and stress signaling.^68,71^

The clearest sign of where the field is heading is co-doping. N,S-, N,P-, Mg,N-, and Ce, N-doped systems combine two dopant effects within a single nanoplatform, producing synergistic gains in light harvesting, charge transfer, antioxidant regulation, nutrient acquisition, and stress resilience.^67,74,93^ This shift from single-element doping toward deliberately combined functionality marks the transition from single-purpose nanomaterials to genuinely multifunctional photosynthetic nano-regulators. Heteroatom doping is, in short, the mechanism by which structural engineering becomes biological specificity. The next section draws size, surface chemistry, and doping together into a single set of design principles for a photosynthetic nano-regulator.

### 3.5 Design principles for photosynthetic nano-regulator

No single physicochemical property determines CQD agricultural performance. Rational design, in contrast, requires treating precursor composition, particle size, surface chemistry, electronic structure, and heteroatom engineering as interacting variables that jointly govern uptake, intracellular transport, chloroplast accessibility, photosynthetic regulation, and stress adaptation. Four design principles capture how these variables interact in practice.

Optical design comes first. CQDs with high quantum yield, broad absorption, and efficient wavelength conversion can increase the amount of photosynthetically active radiation reaching the leaf. Algae-derived, spectral-converting CQDs have demonstrated exactly this, increasing photosynthetic activity and biomass by converting ultraviolet radiation into wavelengths chlorophyll uses more efficiently.^69,94^ Biomass-derived artificial antenna systems demonstrate that excitation-energy transfer can be deliberately engineered rather than discovered incidentally.^70^

Electronic design follows closely. Graphitic domains, defect-rich structures, and heteroatom incorporation jointly set charge mobility and electron-transfer behavior, and therefore a CQD's capacity to interact with the photosynthetic electron-transport chain. Nitrogen-doped and graphitic CQDs exhibit enhanced electron-transfer capacity, and direct chloroplast studies confirm the downstream effect: increased ATP and NADPH production following CQD treatment.^65–67^ Properly engineered, a CQD functions as an active regulator of photosynthetic energy conversion rather than a passive additive.

Redox design addresses a separate constraint. Environmental stress so often impairs photosynthesis through ROS accumulation that antioxidant capacity is not optional for agricultural CQDs operating under field conditions. Selenium-, sulfur-, iron-, and cerium-containing systems all demonstrate ROS-scavenging capacity and photoprotection.^58,72,75^ Nanozyme-like CQDs capable of catalytic ROS detoxification represent the current frontier for sustaining photosynthetic efficiency under prolonged stress.

**Fig. 1:**
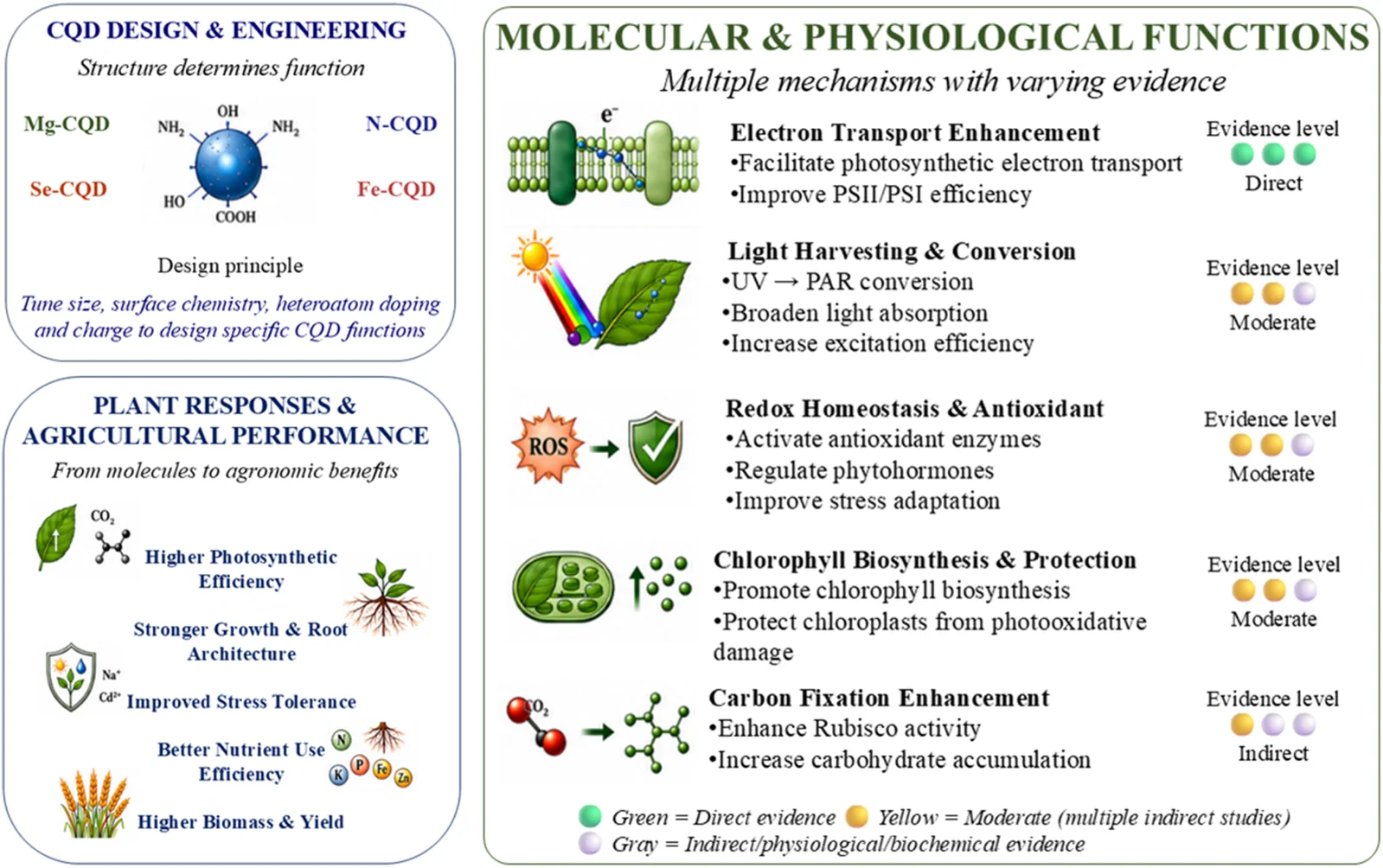
Structure-function-performance framework of CQDs in photosynthetic regulation and sustainable agriculture.

Transport design underlies all three. Particle size, surface functionality, hydrophilicity, and charge jointly determine whether a CQD reaches chloroplast-rich tissue. Optical and electronic sophistication is irrelevant to a particle that never arrives, so biological accessibility belongs in the same design conversation as optical and electronic performance, not as a downstream delivery problem. The most effective systems described in the recent literature integrate all four principles rather than excelling at one. Beyond light harvesting and electron transfer, advanced CQDs regulate nutrient acquisition, carbon metabolism, antioxidant defense, and stress-responsive signaling simultaneously.^71,76,85^ The transcriptomic and metabolomic evidence supporting this pattern suggests that coordinating multiple physiological processes, rather than maximizing any single one, is what distinguishes the strongest-performing CQDs from the rest. Fig. 1 summarizes the integrated structure-function-performance relationships governing CQD-mediated photosynthetic regulation and agricultural performance. Nevertheless, establishing quantitative structure-function-performance relationships that directly correlate CQD design with biological responses remains a major challenge for the field. Establishing them will be essential for moving from descriptive design principles to predictive, application-specific nanotechnology. Section 4 takes the necessary first step toward that goal by examining how these engineered nanomaterials actually enter, move through, and localize within plant tissue: the transport problem that any design principle must ultimately satisfy.

## 4 Uptake, transport, and intracellular localization of CQDs in plants

A CQD's intrinsic physicochemical profile only predicts its biological potential. Realizing that potential requires actually entering plant tissue, moving systemically, and reaching intracellular targets associated with photosynthesis and metabolism. CQDs are well-equipped for this journey: their ultrasmall size, tunable surface chemistry, and high aqueous dispersibility enable them to cross biological barriers efficiently. Uptake efficiency, transport behavior, and final localization still depend heavily on particle size, surface functionality, charge, plant species, developmental stage, and application method.^32,53,95^ This section traces that journey from entry to destination, since the mechanistic link between CQD design and agricultural performance runs directly through it.

### 4.1 Entry pathways into plant systems

**Fig. 2:**
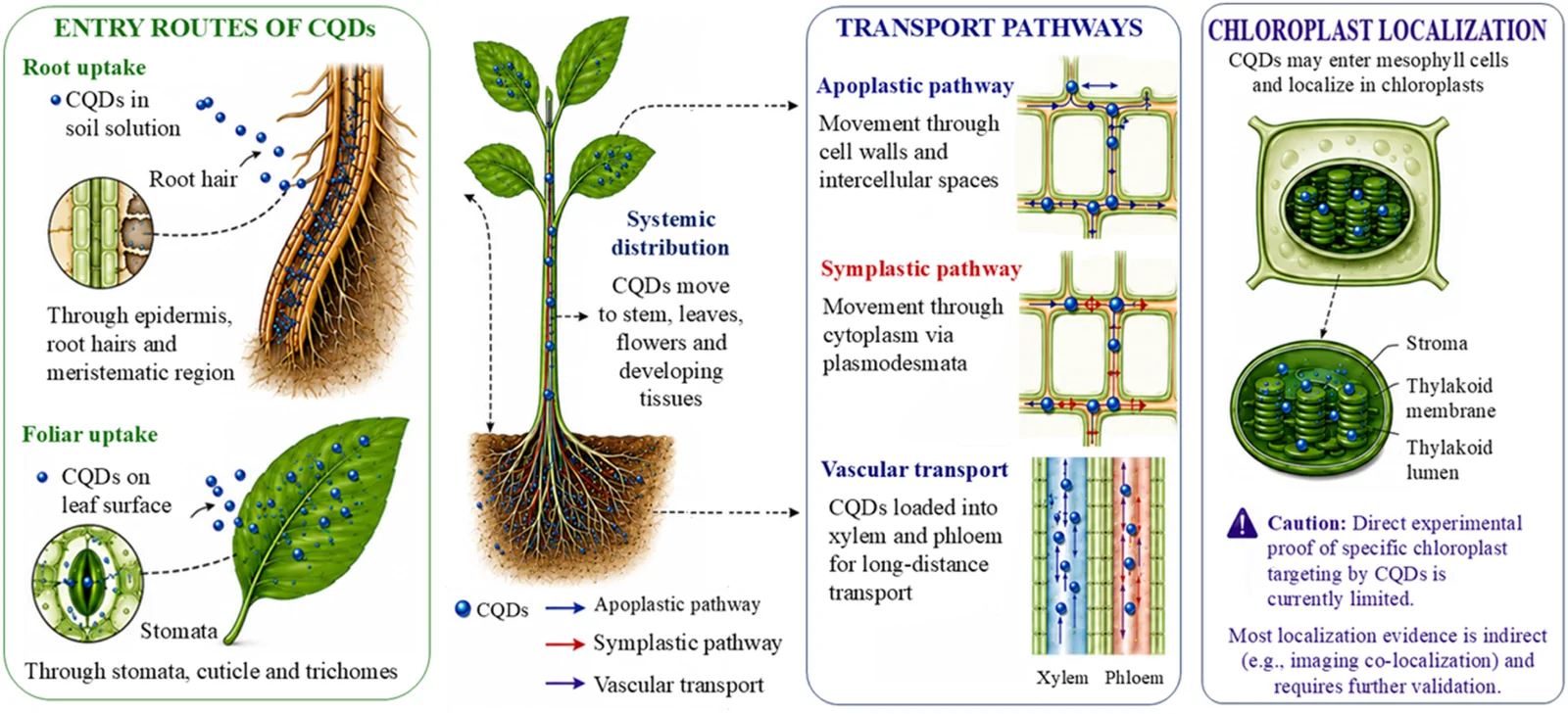
Schematic representation of CQD uptake, vascular transport, chloroplast localization, and their role in enhancing photosynthesis, growth, and stress resilience in plants.

Plants can take up CQDs through several routes: seed priming, root uptake, hydroponic exposure, soil application, and foliar spraying. In each case, nanoscale dimensions and favorable surface chemistry enable the particle to cross the relevant biological barrier and reach internal, metabolically active tissue.^46,48^ Following application, CQDs enter plants through multiple pathways, undergo vascular transport, and localize within chloroplasts, as illustrated in Fig. 2.

Seed priming is the most extensively studied of these routes. CQDs diffuse through seed-coat pores and microchannels, thereby enhancing water uptake and activating the metabolic processes that drive germination and early seedling establishment.^49,96^ The agronomic record is consistent across species: improved germination percentage, seedling vigor, chlorophyll accumulation, and stress tolerance have all been reported after CQD nano-priming in rice, lemon balm, fenugreek, and other crops.^76,83,86,97^

Root uptake is the most mechanistically detailed pathway. CQDs adsorb onto root surfaces, then move through the epidermal and cortical tissues before entering the vascular system, which is responsible for long-distance transport.^98,99^ Their small size allows them to pass through pores in cell walls and intercellular spaces along the way.^49,50^ This behavior is not unique to CQDs. Size, surface chemistry, and colloidal stability govern bioavailability and tissue penetration for engineered nanomaterials in general,^100–103^ confirming that CQDs follow well-established physical rules for nanoparticle uptake rather than a CQD-specific exception.

Foliar application offers the most direct route to photosynthetically active tissue. CQDs deposited on leaf surfaces can enter through stomata, cuticular pathways, or epidermal discontinuities before reaching mesophyll and vascular tissue.^20,104^ Successful foliar delivery has been linked to enhanced photosynthesis, biomass accumulation, and stress resilience in basil, tomato, maize, and rice.^68,69,105^ The practical implication across all three routes is the same: CQD delivery is not constrained to a single application method, which provides considerable latitude to match delivery strategy to crop, growth stage, and target tissue.

### 4.2 Apoplastic and symplastic transport

Once inside plant tissue, CQDs move through two complementary routes. The apoplastic pathway carries them through cell walls and intercellular spaces without crossing plasma membranes. The symplastic pathway carries them cell-to-cell through plasmodesmata.^52,99^

Pore size sets the basic constraint. Plant cell walls typically contain pores of roughly 5 to 20 nm in diameter, large enough for appropriately sized CQDs to pass through root cortical tissue toward the vascular system.^51,52^ Surface chemistry then determines which route a given CQD actually favors. Hydrophilic functional groups improve mobility within the aqueous apoplast, while amino-rich or positively charged surfaces favor membrane interaction and symplastic entry instead.^49,53^

Direct visualization of these pathways is scarce, so evidence of efficient tissue distribution is largely inferential. Improved germination, photosynthesis, nutrient acquisition, biomass, and stress resilience following CQD treatment are consistent with successful transport from the application site to distant metabolically active tissues, but they do not directly prove this.^69,76,86^ Quantifying the relative contribution of apoplastic *versus* symplastic transport will require direct evidence that physiological outcomes alone cannot provide, particularly from advanced imaging, fluorescence tracking, and isotope labeling.

### 4.3 Long-distance vascular transport

CQDs can travel considerable distances through the plant vascular system beyond local tissue movement. Xylem transport carries water and dissolved substances upward from the roots to the shoots along the transpiration stream. Phloem transport redistributes material between source and sink tissues.^20,52,100^ Access to these pathways is directly relevant to this review's central claims, since many of the photosynthetic and stress-resilience effects discussed later occur in tissues well removed from the original application site.

Direct detection confirms that this access exists. CQDs applied at the root have been recovered in stems, leaves, and reproductive organs, demonstrating effective systemic translocation.^49,50^ Transport efficiency is governed by the same physicochemical factors already discussed, namely particle size, hydrophilicity, and surface charge, rather than by any separate set of rules for long-distance movement.^51,53^

Whole-plant physiological responses provide a second, independent line of evidence. Localized CQD treatment routinely produces systemic improvements in photosynthesis, biomass, nutrient utilization, and stress adaptation, which are difficult to explain unless the CQDs themselves or the signals they trigger move efficiently through the plant. Foliar application of red-emissive CDs enhanced photosynthesis, delayed senescence, and increased biomass by 19.81% in tomato.^68^ Algae-derived CQDs raised photosynthetic efficiency and biomass by similarly large margins in basil after foliar treatment.^69^ CQD-mediated gains in nutrient acquisition and drought adaptation have been reported across several other crop systems.^71,85^ What remains unresolved is the quantitative side of this picture. Transport kinetics, tissue-specific accumulation, persistence, and redistribution across developmental stages remain poorly characterized. Fluorescence imaging, isotopic labeling, and high-resolution tracking will be needed to convert today's largely inferential evidence into predictive models of CQD fate within the vascular system, models that will, in turn, be necessary for designing CQDs with reliable delivery efficiency rather than delivery that happens to work in a given experimental system.

### 4.4 Intracellular localization and chloroplast interactions

Once inside a cell, CQDs may localize to the cytoplasm, vacuoles, mitochondria, or chloroplasts, each of which offers a different point of contact with metabolic and signaling machinery.^52,53,55^ Chloroplasts matter most for this review's purposes, since they host light harvesting, photosynthetic electron transport, ATP and NADPH generation, and carbon fixation: the four processes that chloroplast-associated CQD interaction would most plausibly explain.

The earliest evidence for chloroplast-related activity came from nanomaterials research, which showed that engineered particles could improve light utilization and photosynthetic performance by directly interacting with the photosynthetic apparatus.^55,56^ Chandra *et al.*^65^ provided considerably stronger evidence, demonstrating that amine-functionalized CQDs directly enhanced photosynthetic electron transport, ATP production, and NADPH generation in isolated chloroplasts. This work used an isolated chloroplast system rather than relying on whole-plant inference, which is exactly why it remains one of the most convincing direct demonstrations of CQD-photosystem interaction in the literature.

More recent studies have built outward from this foundation. Algae-derived and coffee-ground-derived CQDs further extended this by using wavelength conversion and antenna-like excitation-energy transfer, respectively, to substantially increase photosynthetic efficiency and sugar accumulation in basil and rice;^69,70^ Section 5.1 details the magnitude of these gains, along with the spectroscopic and computational evidence supporting each mechanism. Biochar-derived CQDs increased CO_2_ assimilation by 58% and Rubisco activity by 33% in green mustard,^106^ extending the chloroplast-interaction evidence base to carbon fixation itself, not electron transport alone.

Transcriptomic data add a molecular layer to this picture. Red-emissive CDs promoted photosynthetic activity and delayed senescence in tomato while measurably shifting expression of photosynthesis-related genes.^68^ Comparable improvements in nutrient acquisition, photosynthetic efficiency, and biomass reported in pakchoi point in the same direction:^85^ CQDs appear to act on chloroplast-centered physiology at the gene-expression level, not only at the level of measurable physiological output.

What this evidence base still lacks is direct visualization. Physiological, biochemical, and molecular data all point to chloroplast localization, but no study has yet imaged CQDs unambiguously within an intact chloroplast under conditions that rule out artifacts or contamination. Closing that gap through advanced fluorescence imaging, correlative microscopy, and subcellular tracking is a precondition for treating chloroplast targeting as an established mechanism rather than a well-supported inference. Advanced fluorescence imaging studies have further demonstrated the utility of CQDs for tracing nanoparticle transport and intracellular localization in plant tissues, providing valuable insight into nano-plant interactions.^146^

### 4.5 Concentration-dependent uptake and biological responses

CQD biological activity is concentration-dependent in a fairly predictable way. Low-to-moderate doses tend to enhance germination, photosynthesis, nutrient acquisition, biomass, and stress tolerance, whereas excessive concentrations reduce efficacy or disrupt plant physiology.^47,107^ This pattern reflects a balance familiar in nanotoxicology: beneficial nano-bio interactions at low exposure give way to disrupted cellular homeostasis at high exposure, a pattern not unique to CQDs. The optimal dosage window is real but narrow, and it does not generalize across systems. CQDs improved germination, antioxidant activity, and seedling establishment in lemon balm, with the most effective concentration at 100 mg L^−1^, while both lower and higher concentrations underperformed.^97^ Lawn-waste-derived CQDs instead peaked in salinity mitigation for bermudagrass at roughly 160 mg L^−1^.^84^ Comparable dose-dependent patterns in photosynthesis, nutrient uptake, and biomass are observed in green mustard and pakchoi at other concentrations.^85,106^ The variation across these optimal concentrations is the more important finding for translation, not merely their existence, because it means dosage cannot be assumed to transfer from one CQD-crop combination to another.

CQD composition, particle size, surface chemistry, heteroatom content, plant species, developmental stage, and application route all shift the optimal range. This is precisely why results from one CQD system cannot be extrapolated to another without direct testing. Establishing quantitative dose-response relationships, ideally as a standard reporting requirement, remains a prerequisite for translating laboratory findings into reliable agricultural practice. Across entry, transport, localization, and dosage, the evidence converges on a single conclusion. CQD biological performance is not a fixed property of the particle but an outcome that depends jointly on structural design and the specific conditions of plant exposure. Section 4.6 explicitly draws these structural and transport threads together before Section 5 turns to the mechanisms by which CQDs that do reach the chloroplast actually regulate photosynthesis.

### 4.6 Integrating structural design with transport and photosynthetic performance

The preceding sections have treated structural engineering (Section 3) and transport behavior (Sections 4.1–4.5) sequentially, but the two are mechanistically inseparable. Particle size establishes the first link: CQDs in the 2–10 nm range can cross cell-wall pores and enter vascular pathways (Sections 3.3, 4.2), potentially reaching meristematic and mesophyll tissues where chloroplasts are concentrated. However, a CQD optimized for light harvesting or electron transport (Sections 5.1, 5.2) cannot exert these functions if its size restricts access to target tissues; conversely, a CQD capable of reaching chloroplast-rich cells requires appropriate electronic and redox properties to interact with the photosynthetic apparatus. Size primarily influences access, whereas electronic structure determines functional activity after access.

Surface chemistry and charge provide a second link by influencing intracellular accessibility. Amino-functionalized, positively charged CQDs favor membrane interaction and symplastic entry (Sections 3.3, 4.2), potentially increasing access to intracellular and chloroplast-associated targets. Hydroxyl- and carboxyl-rich, negatively charged CQDs generally favor aqueous mobility and apoplastic transport, which may support systemic distribution rather than direct chloroplast-level interaction. Thus, surface chemistry selected for colloidal stability or long-distance mobility can influence membrane affinity and intracellular access, creating an important design consideration for the photosynthetic mechanisms discussed in Sections 5.1 and 5.2.

Heteroatom doping provides a further connection because it can influence both transport and subsequent activity. Nitrogen and amino-group incorporation can modify surface charge and hydrophilicity, affecting membrane interaction and uptake, while nitrogen doping also alters electron density and conductivity within the CQD core. These electronic changes are associated with enhanced electron transport documented in isolated chloroplasts.^65,67,87^ Magnesium and cerium doping provide related examples in which material composition is associated with chlorophyll-related and photoprotective functions,^73,74,92^ whereas iron doping contributes primarily through nanozyme-like catalytic activity requiring sufficient intracellular access.^75^ Thus, doping should be considered a mechanism-specific design variable rather than a uniform enhancement strategy.

Taken together, these relationships indicate that photosynthetic performance depends on the coupling of access and action, rather than on independent optimization of individual properties. A CQD with favorable electronic properties but limited access to chloroplast-rich tissues may provide little biological benefit, whereas efficient access without appropriate optical, electronic, or redox functionality may likewise be ineffective. The comparative relationships among CQD physicochemical traits, plant accessibility, photosynthetic targets, and evidence strength are summarized in Table 3. The strongest evidence across Sections 4 and 5, including nitrogen-doped systems associated with electron transport,^65,67,87^ algae- and coffee-derived systems associated with light harvesting,^69,70^ and multi-element systems affecting multiple processes,^67,74,93^ therefore supports an integrated structure-function-performance framework. Section 5 examines these photosynthetic mechanisms in detail.

**Table 3:** Comparative mapping of CQD physicochemical traits to plant accessibility, photosynthetic mechanisms, biological consequences, and evidence strength^a^

S. No.	Physicochemical trait	Representative CQD design	Primary physicochemical effect	Plant accessibility/uptake	Photosynthetic target/mechanism	Biological consequence	Evidence strength	References
1	Particle size	2–10 nm CQDs	High surface area and nanoscale mobility	Facilitates interaction with cell walls, intercellular spaces, and vascular pathways; effects on chloroplast access remain largely inferential	Photosynthetic machinery; size primarily influences access rather than the reaction itself	Greater tissue distribution and potential photosynthetic regulation; excessive reduction in size may also affect retention	Moderate/inferential	49–53
2	Positive surface charge	Amine-functionalized CQDs	Enhanced electrostatic interaction with negatively charged plant surfaces	Promotes membrane association and cellular uptake	Photosynthetic electron transport	Increased electron-transfer activity; amine-CQDs produced ∼93% enhancement in isolated chloroplasts and 26% higher Hill activity	Direct	52, 53 and 65
3	Negative surface charge	Carboxyl-/hydroxyl-rich CQDs	High aqueous dispersibility and reduced membrane affinity	Favors aqueous/apoplastic transport; systemic distribution may occur depending on surface chemistry	Photosynthetic regulation	Potentially broader tissue distribution; direct evidence for chloroplast-level action remains limited	Moderate/indirect	32, 52, 53 and 79
4	Nitrogen doping	N-CQDs; pyridinic/pyrrolic/graphitic N	Modified electronic structure, electron density, and charge mobility	Surface N groups can also modify hydrophilicity and plant interaction	Electron transport and energy conversion	Enhanced photosynthesis, carbon assimilation, and carbohydrate accumulation; photosynthesis increased by 21.5% in maize and ∼24% in apple	Strong/moderate-to-direct	66, 67 and 87
5	Phosphorus doping	P-CQDs; N,P-CQDs	Modified charge distribution and charge-separation behavior	Surface and electronic properties may influence intracellular activity	Electron transfer and photochemical conversion	Improved photochemical efficiency and potential enhancement of carbon assimilation	Moderate	58 and 81
6	Sulfur doping	S-CQDs; N,S-CQDs	Altered surface states and redox activity	Provides redox-active functionality following uptake	ROS homeostasis and photoprotection	Enhanced ROS regulation and preservation of photosynthetic machinery, particularly under stress	Moderate	89 and 145
7	Selenium doping	Se-CQDs; Se,N-CQDs	Redox-active and stress-responsive surface/electronic states	Supports intracellular antioxidant and stress-response processes	ROS regulation and stress-adaptive photosynthesis	Improved antioxidant defense, photosynthetic resilience, and salinity tolerance	Moderate	58, 72, 90 and 91
8	Magnesium doping	Mg-CQDs; Mg,N-CQDs	Modifies optical properties and introduces chlorophyll-associated functionality	May improve interaction with chloroplast-associated processes	Light harvesting and photosystem stability	Improved light utilization, chlorophyll stability, and photosynthetic resilience under salinity or UV-B stress	Moderate	73 and 74
9	Iron doping	Fe-CQD nanozyme	Introduces enzyme-mimetic catalytic redox activity	Requires access to sites of ROS generation	ROS homeostasis and photoprotection	Catalytic ROS scavenging and sustained protection of photosynthetic machinery under oxidative stress	Moderate	75

a Abbreviations: CQD, carbon quantum dot; N-CQD, nitrogen-doped carbon quantum dot; N,P-CQD, nitrogen/phosphorus co-doped carbon quantum dot; N,S-CQD, nitrogen/sulfur co-doped carbon quantum dot; Se-CQD, selenium-doped carbon quantum dot; Mg-CQD, magnesium-doped carbon quantum dot; Mg,N-CQD, magnesium/nitrogen co-doped carbon quantum dot; Fe-CQD, iron-doped carbon quantum dot; ROS, reactive oxygen species; Hill activity, photosynthetic electron-transfer activity measured using the Hill reaction. Evidence strength: direct, experimentally demonstrated mechanistic interaction; moderate, supported by biochemical, photophysical, physiological, or molecular evidence but lacking definitive target-level validation; indirect, inferred mainly from downstream physiological responses.

## 5 Structure-function-performance relationships in photosynthetic nano-regulator

Photosynthesis sets the ceiling on plant productivity, biomass accumulation, and resource-use efficiency, which is why any nanomaterial that can improve it draws disproportionate agricultural interest. CQDs differ from most agricultural nanomaterials in this respect. Instead of acting as nutrient carriers or generic growth stimulants, they possess optical, electronic, and redox properties that enable them to interact directly with multiple components of the photosynthetic apparatus.^55,62,67^

**Fig. 3:**
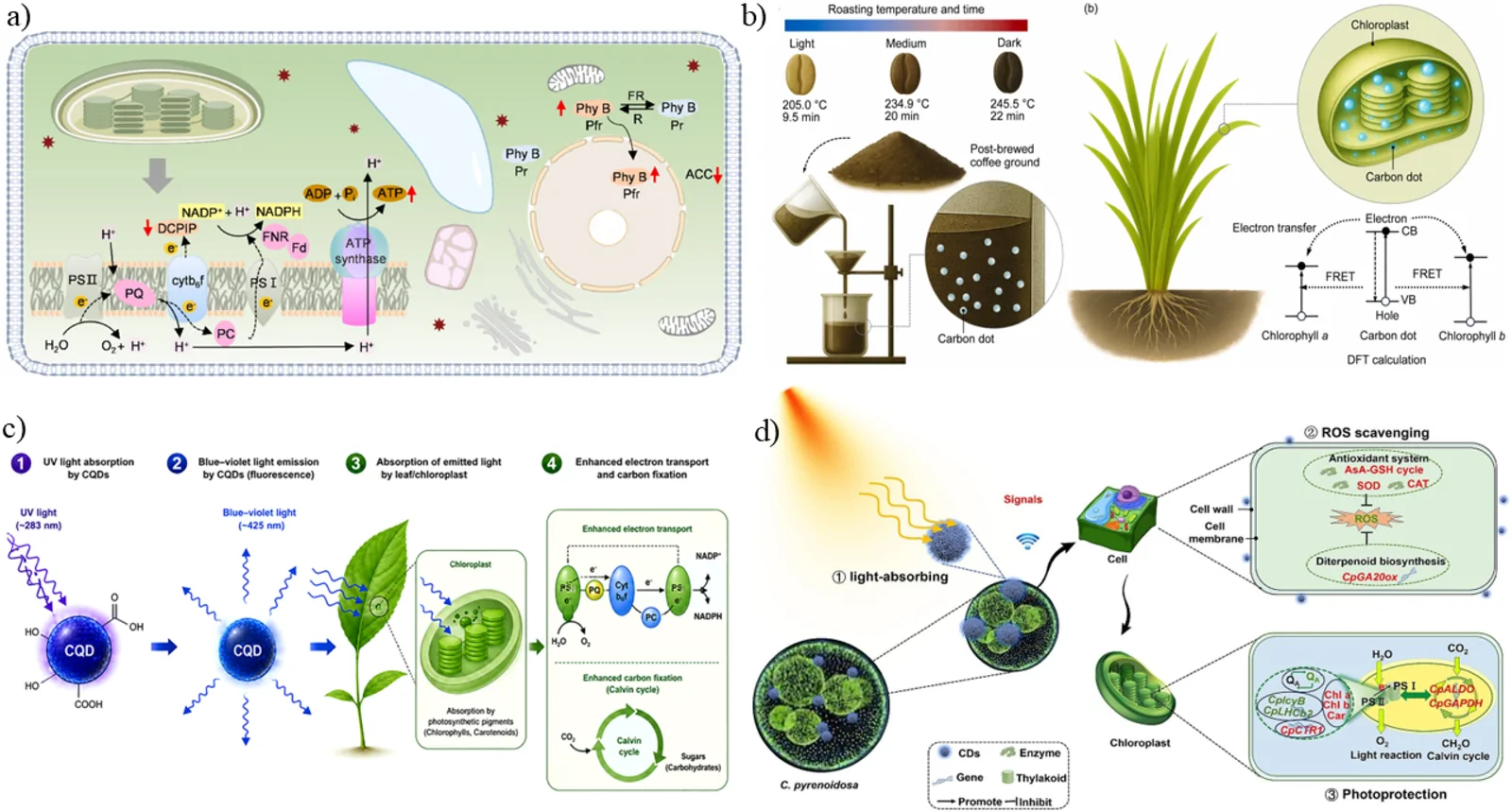
Representative mechanisms by which CQDs enhance photosynthesis and photoprotection in plants. (a) CQD-mediated regulation of photosynthetic electron transport, ATP/NADPH production, and phytochrome signaling. (b) Coffee ground-derived CQDs enhance light harvesting and facilitate electron transfer in chloroplasts. (c) CQDs improve light utilization by converting UV light into blue fluorescence, thereby enhancing electron transport and carbon fixation. (d) CQD-induced photoprotection through ROS scavenging, antioxidant defense, and regulation of photosynthetic pathways. Reproduced with permission from *Journal of Plant Physiology*, Elsevier (License No. 6300600679322) (a); *Industrial Crops and Products*, Elsevier (License No. 6300591405976) (b); adapted from ref. 69 under the Creative Commons Attribution 4.0 International (CC BY 4.0) License (c); and *Chemical Engineering Journal*, Elsevier (License No. 6300600881505) (d).

That interaction operates through five interconnected mechanisms: enhancement of photosynthetic electron transport, light harvesting and spectral conversion, redox homeostasis and antioxidant regulation, chlorophyll biosynthesis and photoprotection, and carbon fixation and metabolic regulation. The evidence supporting these mechanisms is not uniform: direct chloroplast-level evidence is strongest for electron-transport enhancement, whereas the remaining mechanisms are supported predominantly by physiological, biochemical, photochemical, transcriptomic, or metabolic observations.^65–71,108–110^ Treating these five mechanisms as equally well established would overstate what the literature actually shows. The five mechanisms are also not independent of one another. Optical engineering raises photon availability. Improved electron transport raises ATP and NADPH supply. Improved carbon assimilation converts that energy into biomass. Antioxidant regulation protects the machinery doing all of this from environmental stress. Photosynthetic nano-regulator is best understood as the combined output of these interlocking steps, not the work of any single mechanism acting alone. The five sections that follow examine each pathway in turn, organized by the strength of its supporting evidence and weighted toward the structural features responsible for each biological response. Table 4 and Fig. 1 summarize the resulting structure-function-performance relationships and evidence hierarchy. The principal mechanisms by which CQDs regulate photosynthesis, including enhanced light harvesting, electron transport, carbon assimilation, and photoprotection, are summarized in Fig. 3.

**Table 4:** Carbon quantum dots as photosynthetic nano-regulators: mechanistic evidence and structure-function relationships

Category 1 electron transport enhancement												
CQD design	Dopant/functionalization	Plant system	Photosynthetic target	Proposed mechanism	Evidence level^a^				Physiological response	Quantitative outcome	Key contribution	References
Amine-CQD	Surface amination	Isolated chloroplasts	PSI–PSII electron transport chain	CQDs facilitate electron transfer and charge separation, increasing photosynthetic energy conversion	Direct				Increased electron transport, ATP and NADPH generation	Electron transport ↑93%; Hill activity ↑26%	Direct chloroplast-level evidence for CQD participation in photosynthetic electron transport	65
Biomass-derived CQDs	Biomass precursor	*Arabidopsis*	Photosynthetic machinery	Direct photoelectron injection enhances photosynthetic energy conversion	Direct				Increased carbon fixation and biomass	CO_2_ fixation ↑2.4-fold; biomass ↑1.8-fold	Links CQD-mediated electron transfer with whole-plant carbon fixation and biomass production	66
N-CQD	Nitrogen doping	Maize	Photosystem activity	Increased electron availability and charge mobility enhance photosynthetic electron transfer	Moderate				Increased photosynthesis and carbohydrate accumulation	Photosynthesis ↑21.5%; carbohydrates ↑66%	Demonstrates the relationship between N doping, electron transfer, and photosynthetic performance	67
N-CQD	Nitrogen doping	Apple	Plastoquinone pool	Accelerated electron transfer within the photosynthetic electron transport chain	Moderate				Enhanced photosynthesis and fruit quality	Photosynthesis ↑24%	Supports a plastoquinone-mediated route for CQD-enhanced electron transport	87
CQDs	Biochar-derived	Green mustard	Chloroplast electron transport	Enhanced CQD-chloroplast electron-transfer interaction	Moderate				Increased photosynthesis and Rubisco activity	CO_2_ assimilation ↑58%; Rubisco ↑33%	Connects improved chloroplast electron transfer with downstream carbon assimilation	106

**Category 2 light harvesting and conversion**												
Algae-derived CQDs	*Gracilaria corticata*-derived	Basil	Light harvesting and photosynthetic activity	UV-to-visible spectral conversion increases the fraction of incident radiation available for photosynthesis				Moderate	Enhanced photosynthesis and biomass	Photosynthesis ↑44.65%; biomass ↑30%	Demonstrates spectral-converting CQDs as photosynthetic nano-regulators	69
Coffee-ground CDs	Biomass-derived	Rice	Light harvesting and chloroplast electron transport	Förster resonance energy transfer (FRET)-mediated excitation-energy transfer produces an artificial antenna effect				Moderate	Enhanced photosynthesis and sugar accumulation	Photosynthesis ↑32.66%; sugars ↑100.18%	Provides experimental and density functional theory (DFT) support for CQD-based artificial photosynthetic antennae	70
Mg-CQD	Magnesium doping	Rice	Light harvesting and chloroplast function	Mg-associated functionality supports light capture and chloroplast compatibility				Moderate	Improved photosynthesis and salinity tolerance	Significant enhancement reported	Links Mg incorporation with chlorophyll-associated light utilization and photosynthetic stability	73
Mg,N-CQD	Mg/N co-doping	Wheat	Photochemistry and light utilization	Combined Mg-associated photoprotection and N-mediated electronic effects support photosynthetic stability				Moderate	Improved photosynthetic stability under UV-B	Significant enhancement under UV-B	Integrates light utilization with antioxidant and UV-B protection	74
CQD-AIEgens	Aggregation-induced-emission functionalization	Model photosynthetic systems	Solar-energy capture	Enhanced excitation transfer and light harvesting through engineered emissive states				Moderate	Increased photochemical efficiency	Significant enhancement reported	Demonstrates artificial antenna-like CQD behavior	111

**Category 3 redox homeostasis and antioxidant regulation**												
Se,N-CQD	Se/N co-doping	Rice	Stress-adaptive photosynthesis	Ca^2+^- and jasmonate-associated signaling coordinates antioxidant defense and photosynthetic protection		Moderate			Improved salinity tolerance and antioxidant defense	Enhanced antioxidant response	Links heteroatom co-doping with stress signaling and photosynthetic protection	58
Se-CQD	Selenium doping	Rapeseed	Seedling photosynthesis and ROS regulation	Activation of antioxidant defense and ROS-regulatory pathways during germination		Indirect			Improved germination, seedling vigor and salt tolerance	Significant increase in vigor	Demonstrates nano-priming-associated photosynthetic resilience	72
Fe-CQD nanozyme	Iron doping	Lettuce	ROS-regulated photosynthesis	Enzyme-mimetic catalytic ROS scavenging reduces oxidative pressure on photosynthetic machinery		Moderate			Reduced oxidative damage and improved photosynthetic protection	Significant ROS reduction	Introduces sustained nanozyme-assisted photoprotection	75
Carbon dots	Undoped	Cotton	ROS-mediated salt adaptation	NADPH oxidase-dependent H_2_O_2_ signaling contributes to Ca^2+^-associated SOS regulation and Na^+^ homeostasis		Moderate			Improved germination and salt tolerance	Root length ↑205.2%; germination ↑32%; Na^+^ accumulation ↓15.3%	Mechanistically links ROS signaling with ion homeostasis and early seedling establishment	138
Biomass-derived CQDs	Plant-derived	*Chlorella pyrenoidosa*	Photosystem protection	Reduced ROS accumulation alleviates photoinhibition and preserves photosynthetic activity		Moderate			Improved photosynthetic performance	Significant enhancement reported	Extends CQD-mediated photoprotection to photosynthetic microalgae	147

**Category 4 chlorophyll biosynthesis and protection**												
Mg-CQD	Magnesium doping	Rice	Chlorophyll-associated light harvesting and chloroplast function	Mg incorporation supports chlorophyll-associated processes, light utilization, and chloroplast stability	Moderate			Improved chlorophyll-related photosynthetic performance and salinity tolerance		Significant enhancement reported	Provides a biomimetic route linking Mg-containing CQDs with chlorophyll-associated function	73
Mg,N-CQD	Mg/N co-doping	Wheat	Chlorophyll protection and photochemistry	Mg-associated chloroplast compatibility combined with N-mediated electronic and antioxidant effects limits UV-B-associated damage	Moderate			Preserved photosynthetic stability under UV-B stress		Significant enhancement under UV-B	Couples pigment/chloroplast protection with photochemical and antioxidant functions	74
Ce-CQD	Cerium doping	Plant stress systems	Chlorophyll biosynthesis and photochemical performance	Ce-associated redox regulation supports chlorophyll biosynthesis while limiting oxidative impairment of the photosynthetic apparatus	Moderate			Improved chlorophyll status and photosynthetic protection		Significant improvement reported	Links redox regulation with maintenance of photosynthetic pigments	92
N-CQD	Nitrogen doping	Rice	Chlorophyll and photosynthetic machinery under arsenic stress	Enhanced antioxidant defense limits oxidative damage and preserves chlorophyll under heavy-metal stress	Moderate			Improved chlorophyll content and photosynthetic performance		Chlorophyll ↑59%; as accumulation ↓58%	Demonstrates pigment preservation as a component of CQD-mediated photoprotection	86

**Category 5 carbon fixation enhancement**												
CQDs	Undoped	Rice	Rubisco and calvin cycle	CQD treatment enhances carbon fixation and Rubisco activity, increasing conversion of assimilated CO_2_ into biomass			Indirect		Increased productivity	Rubisco ↑42%; grain yield ↑14.8%	First crop-scale evidence connecting CQDs with enhanced carbon assimilation	121
Glucose-CQD	Glucose functionalization	Wheat	Whole-plant photosynthesis and carbon assimilation	Improved carbon assimilation supports photosynthetic productivity and yield			Indirect		Enhanced photosynthesis and productivity	Significant increase in photosynthesis and yield	Demonstrates crop-scale productivity enhancement through carbon assimilation	122
Orange CQDs	Plant-derived	Green bean	CO_2_ assimilation	Enhanced chloroplast activity supports carbon fixation downstream of CQD-mediated photochemical effects			Indirect		Improved photosynthetic performance	Significant increase in CO_2_ assimilation	Links engineered optical CQDs with enhanced carbon assimilation	94
CQDs	Biomass-derived	Soybean	Photosynthesis and carbon–nitrogen metabolism	Improved photosynthetic activity is coordinated with amino-acid biosynthesis and nitrogen metabolism under drought			Indirect		Improved drought tolerance and photosynthetic performance	Significant enhancement reported	Demonstrates coupling between carbon assimilation and nitrogen metabolism	112
Chiral CQDs	Surface chirality	Mung bean	Rubisco activity	CQD chirality modulates Rubisco-associated carbon fixation			Moderate		Enhanced photosynthesis	Rubisco ↑21–68%	Demonstrates stereochemical control of photosynthetic carbon fixation	148

a Evidence level: direct = mechanistic evidence obtained from chloroplast-level studies, isolated photosynthetic systems, or direct electron/photoelectron-transfer investigations; moderate = mechanism supported by physiological, biochemical, transcriptomic, photochemical, pharmacological, or molecular evidence; indirect = mechanism inferred primarily from photosynthetic performance, growth, biomass, germination, stress tolerance, or yield responses. Evidence levels were assigned based on the degree of mechanistic validation reported in the cited studies and are intended to facilitate comparison among studies rather than to evaluate their overall scientific quality. Abbreviations: CQD, carbon quantum dot; ROS, reactive oxygen species; FRET, Förster resonance energy transfer; PSI, photosystem I; PSII, photosystem II; PETC, photosynthetic electron transport chain; Rubisco, ribulose-1,5-bisphosphate carboxylase/oxygenase; AIE, aggregation-induced emission.

### 5.1 Photosynthetic electron transport enhancement

Of the five mechanisms surveyed in this section, facilitation of photosynthetic electron transport carries the strongest direct experimental support, and for good reason. Efficient electron transfer through the photosynthetic electron transport chain (PETC) generates ATP and NADPH, which ultimately drive carbon fixation, biomass production, and growth. A material that measurably improves electron-transfer efficiency, therefore, has a plausible, mechanistically direct route to improving everything downstream. Chandra *et al.*^65^ supplied the first such direct evidence, showing that amine-functionalized CQDs raised electron-transfer activity in isolated chloroplasts by approximately 93%, alongside a 26% increase in Hill-reaction activity and corresponding gains in ATP and NADPH production. The experiment was conducted in isolated chloroplasts, not whole plants, so it directly demonstrates CQD's participation in photosynthetic energy conversion rather than inferring it from downstream physiological outcomes. This is exactly why it remains the benchmark study for this mechanism.

Subsequent work has extended this finding across plant systems rather than merely replicating it. Nitrogen-doped CQDs raised photosynthetic performance by 21.5% and carbohydrate accumulation by 66% in maize, attributed to increased electron availability and more efficient photosystem activity.^67^ N-CQDs increased photosynthesis by approximately 24% in apple through accelerated electron transfer within the plastoquinone pool.^87^ The quantitative outcomes differ by crop, but the underlying mechanism is consistent: heteroatom doping reshapes charge-transfer behavior within the PETC. The strongest recent evidence directly links this electron-transport effect to carbon fixation. Cheng *et al.*^66^ showed that engineered, biomass-derived CQDs that facilitate photoelectron transfer in *Arabidopsis* increased CO_2_ fixation by 2.4-fold and biomass by 1.8-fold, effectively closing the mechanistic loop from electron transport to whole-plant productivity. Biochar-derived CQDs reinforce the same loop in green mustard, where improved electron transport accompanied a 58% increase in CO_2_ assimilation and a 33% increase in Rubisco activity.^106^ These effects trace back to identifiable structural features. Graphitic domains support charge mobility. Surface defects and heteroatom incorporation create electronic states that improve charge separation and electron-transfer efficiency.^30,33^ Nitrogen doping is particularly effective because it directly increases electron density and conductivity, promoting electron migration through the photosynthetic apparatus.^67,87^ Phosphorus-containing and co-doped CQDs may offer similar advantages by reducing electron–hole recombination, though direct evidence for this specific claim in plant photosystems is still limited.^58,81^ Among the five mechanisms considered here, electron-transport enhancement has the strongest mechanistic support because it is supported by direct intervention in an isolated chloroplast system. The reported enhancement of electron-transfer activity provides the clearest causal connection between CQD electronic structure and photosynthetic function, although the precise interaction sites within PSI and PSII remain unresolved.^65,66,87^

### 5.2 Light harvesting and energy conversion

Improved light harvesting is among the oldest and most intuitive mechanisms proposed for CQD-mediated photosynthetic enhancement, and for good reason. Conventional photosynthetic pigments use only a fraction of the solar spectrum, leaving substantial incident energy photochemically unused.^55,56^ CQDs, with their tunable absorption and photoluminescence, are well suited to recovering some of that unused energy through nanoscale spectral management. Their key trick is wavelength conversion: absorbing high-energy ultraviolet photons and re-emitting them as visible light in spectral regions chlorophyll uses more efficiently. Algae-derived CQDs have a direct agronomic effect, increasing basil photosynthetic performance by 44.65% and biomass by 30% through UV-to-visible conversion and improved light utilization.^69^ Orange-emissive CQDs produced comparable gains in photosynthetic activity and carbon assimilation in green bean.^94^ Some CQDs function as artificial light-harvesting antennae outright, beyond simple wavelength conversion. Coffee-ground-derived CDs increased photosynthesis by 32.66% and soluble sugar accumulation by 100.18% in rice, with spectroscopic data and density functional theory calculations together supporting excitation energy transfer between the CQDs and the photosynthetic apparatus.^70^ This is essentially the same physics that aggregation-induced-emission CQD systems exploit when acting as artificial antennae in other contexts.^111^

Effectiveness here is a direct function of structural design. High quantum yield, broad absorption, well-matched emission wavelengths, and efficient charge separation all determine the spectral conversion and energy transfer a given CQD can deliver. Magnesium-containing systems may offer an additional advantage through improved chloroplast compatibility and photoprotection.^73,74^ The evidence base here is real but still indirect. Most of it comes from whole-plant physiological measurements and photophysical analyses, not from direct chloroplast imaging of the proposed mechanism in action. That caveat does not undermine the conclusion so much as bind it. Optical engineering remains one of the most promising routes to next-generation photosynthetic nano-regulators, but a promising mechanism and a directly demonstrated one are not the same claim. Thus, CQDs can function as spectral converters or artificial light-harvesting components, increasing the fraction of incident radiation available to photosynthetic pigments; however, evidence for direct CQD-photosystem energy transfer inside intact chloroplasts remains more limited than the evidence for electron-transport enhancement.

### 5.3 ROS homeostasis and antioxidant regulation

Photosynthetic efficiency under field conditions is rarely limited by light or CO_2_ alone. Drought, salinity, temperature extremes, heavy metals, and excess ultraviolet radiation routinely disrupt electron transport, destabilize pigments, and trigger excess reactive oxygen species (ROS) production, with oxidative damage and reduced productivity following close behind. Sustaining photosynthesis under stress is therefore as much a redox problem as a photochemical one.

CQDs address this redox problem differently from conventional antioxidants. Engineered CQDs can simultaneously modulate photochemistry, redox signaling, and stress-responsive metabolism, rather than simply scavenging ROS. This multifunctional capability allows them to protect photosynthesis while maintaining metabolic activity under adverse conditions, rather than merely limiting damage after the fact. Selenium doping illustrates this well. Se-containing CQDs activate antioxidant enzymes, strengthen stress-responsive signaling, and maintain photosynthetic activity under salinity stress.^72,90^ Se,N-co-doped systems extend the same protective effects to ion homeostasis and oxidative balance simultaneously,^91^ providing evidence that heteroatom engineering can broaden CQD function from photosynthetic enhancement to general physiological protection. Sulfur doping operates *via* a related yet distinct route, enhancing ROS-scavenging activity and antioxidant capacity directly,^89^ thereby protecting chloroplast membranes and photosynthetic proteins from oxidative damage. Magnesium- and Mg,N-co-doped CQDs enhance chloroplast stability and UV-B tolerance within this protective profile,^73,74^ underscoring how element-specific engineering can be tailored to the specific stress a crop is expected to face. Iron-doped CQDs introduce a structurally distinct solution: catalytic protection rather than consumable protection. Their nanozyme-like, enzyme-mimetic activity continuously scavenges ROS rather than being depleted by a single detoxification event,^75^ a property that makes them attractive for sustained protection in field conditions where stress exposure is prolonged rather than episodic. Cerium-containing CQDs add a complementary redox role, supporting chlorophyll biosynthesis alongside general photoprotection.^92^

The protective effect of CQDs extends beyond direct antioxidant action into broader stress-adaptation networks. Nitrogen-doped CQDs improved arsenic tolerance in rice by maintaining chlorophyll content and reducing oxidative damage.^86^ CQD priming improved drought resilience in *Elymus sibiricus* by coordinating the regulation of photosynthesis, carbohydrate metabolism, and stress-responsive pathways.^71^ Transcriptomic and metabolomic data reinforce the same conclusion: engineered CQDs modulate genes involved in ROS signaling, energy metabolism, ion transport, and environmental adaptation,^68,71,76^ confirming that their protective effect extends well beyond simple ROS scavenging. Biomass-derived CQDs have also alleviated photoinhibition in *Chlorella pyrenoidosa* by reducing ROS accumulation and improving photosynthetic performance, demonstrating that CQD-mediated photoprotection extends beyond higher plants to photosynthetic microorganisms.^147^ As with the other mechanisms in this section, photoprotective performance stems from identifiable structural choices: heteroatom incorporation, defect engineering, surface functionalization, and nanozyme integration. Each can be matched to a specific environmental constraint without sacrificing photosynthetic efficiency or growth. This capacity to combine stress protection with photosynthetic enhancement, rather than trading one off against the other, distinguishes CQDs from most conventional agricultural nanomaterials. Taken together, ROS homeostasis, antioxidant defense, chloroplast protection, and stress-responsive signaling form a coordinated protective system that sustains carbon assimilation and biomass production under conditions where photosynthesis would otherwise fail. For agricultural deployment under realistic, variable field conditions, this protective dimension may matter as much as direct photosynthetic enhancement. Collectively, these findings indicate that CQDs can influence the redox environment surrounding the photosynthetic apparatus through both antioxidant and signaling functions. Nevertheless, most evidence derives from ROS measurements, antioxidant enzyme activities, and stress phenotypes rather than from direct visualization of CQD interactions with chloroplast redox components.

### 5.4 Chlorophyll biosynthesis and protection

Chlorophyll content and chloroplast integrity provide an important link between CQD treatment and sustained photosynthetic performance. Environmental stresses such as salinity, drought, ultraviolet radiation, and heavy-metal exposure can accelerate chlorophyll degradation and impair chloroplast function, thereby limiting light capture and downstream photochemistry. CQDs can counter these effects through element-specific interactions with chlorophyll-associated processes and through protection against oxidative damage. Magnesium-containing CQDs are particularly relevant because Mg is the central metal ion of the chlorophyll molecule; Mg-CQDs have been associated with improved chloroplast compatibility, light harvesting, photosynthetic efficiency, and tolerance to salinity stress.^73^ Mg,N-co-doped CQDs extend this functionality by combining chlorophyll-associated effects with antioxidant and UV-B protective activity, thereby helping maintain photosynthetic stability under radiation stress.^74^ Cerium-containing CQDs provide a complementary mechanism, supporting chlorophyll biosynthesis and photochemical performance through redox-mediated regulation of cellular metabolism.^92^ Protection of chlorophyll is also evident in nitrogen- and selenium-containing systems, where enhanced antioxidant defense limits ROS-mediated damage to chloroplast membranes and photosynthetic pigments.^72,86,90^ Thus, CQD-mediated maintenance of chlorophyll should not be interpreted solely as increased pigment synthesis; it can also result from reduced oxidative degradation and preservation of chloroplast structure. The available evidence therefore supports a coupled mechanism in which element-specific CQD chemistry promotes pigment maintenance, while redox regulation protects the chloroplast environment where photosynthesis occurs. However, most studies infer this relationship from changes in chlorophyll content, fluorescence, and stress markers rather than directly demonstrating CQD interactions with chlorophyll biosynthetic enzymes or chloroplast ultrastructure, thereby placing this mechanism at a moderate level of evidence.

### 5.5 Carbon fixation enhancement

The ultimate photosynthetic consequence of enhanced light capture and electron transport is increased carbon fixation. CQDs have been reported to enhance Rubisco activity, CO_2_ assimilation, carbohydrate accumulation, and, in some systems, crop productivity. These observations provide an important functional link between CQD-mediated photochemical regulation and biomass formation. Light harvesting and electron transport improve photosynthetic energy conversion, but their agricultural value depends entirely on what happens to that energy next, namely, whether it is actually converted into fixed carbon and usable biomass. Carbon assimilation is the mechanistic link between improved photochemistry and agronomic outcomes, and a growing body of evidence shows that CQDs act directly on it, not merely as a downstream consequence of better light capture. Li *et al.*^121^ provided the earliest demonstration of this link, reporting a 42% increase in Rubisco activity and a corresponding 14.8% increase in grain yield in rice following CQD treatment. This showed that CQDs can influence Calvin-cycle activity and carbon fixation beyond their effects on photochemistry alone. Swift *et al.*^122^ extended this conclusion to wheat, where glucose-functionalized CQDs improved photosynthesis and yield by enhancing carbon assimilation. More recent work has quantified the direct connection between electron transport and CO_2_ fixation. The *Arabidopsis* and green-mustard datasets discussed in Section 5.2, in which biomass- and biochar-derived CQDs raised CO_2_ fixation and Rubisco activity severalfold,^66,106^ make the same point from the carbon-metabolism side of the ledger: electron-transport enhancement converts into fixed carbon rather than being dissipated elsewhere in the photosynthetic sequence. Orange-emissive CQDs produced a parallel effect on chloroplast activity and carbon assimilation in green bean.^94^ Additional studies have further supported the role of engineered CQDs in enhancing photosynthetic carbon assimilation by coordinating electron transfer and carbon metabolism.^140^

CQD-mediated carbon assimilation also extends beyond Rubisco activation into nitrogen and amino acid metabolism. Nitrogen-doped CQDs raised carbohydrate accumulation alongside photosynthetic efficiency in maize.^67^ CQD treatment improved both photosynthesis and amino acid biosynthesis in soybean under drought,^112^ evidence that carbon and nitrogen metabolism are regulated together, not independently. Improvements in nutrient acquisition and photosynthetic activity reported in coriander reinforce this point: CQDs appear to coordinate carbon assimilation with nutrient utilization rather than stimulating either in isolation.^113^ The most recent layer of evidence concerns metabolic reprogramming rather than single-pathway stimulation. CQD priming enhanced drought tolerance in *Elymus sibiricus* through coordinated regulation of photosynthesis and carbohydrate metabolism.^71^ Red-emissive CDs delayed senescence and increased tomato biomass while modulating photosynthesis-related pathways at the transcriptomic level.^68^ These studies position CQDs as regulators of metabolic networks, rather than merely photochemical enhancers with downstream metabolic side effects. Structural features explain why some CQDs achieve this, and others do not. Strong electron-transfer capability, efficient light harvesting, and favorable chloroplast interaction together raise ATP and NADPH availability for Calvin-cycle activity. Nitrogen doping, graphitic domains, and optimized surface functionality consistently support this combination.^66,67,87^ Chirality adds a further, less expected variable: chiral CQDs modulated Rubisco activity by 21–68% in mung bean, depending on chirality, demonstrating that stereochemistry, not just elemental composition, can tune photosynthetic performance.^148^ Across these interconnected pathways, electron transport, Rubisco activation, CO_2_ fixation, and coordinated carbon-nutrient metabolism, carbon assimilation functions as the mechanistic bridge that converts CQD-enhanced photochemistry into the biomass gains, productivity improvements, and stress resilience reported throughout the agricultural literature.

### 5.6 Molecular signaling, multi-omics insights, and integrated photosynthetic regulation

The five mechanisms described above are interconnected rather than independent, and multi-omics studies increasingly indicate that CQD activity extends beyond photosynthetic regulation to broader networks that control nutrient acquisition, metabolism, and stress adaptation. Transcriptomic analyses have identified CQD-responsive genes associated with photosynthetic electron transport, chlorophyll biosynthesis, carbon metabolism, antioxidant defense, and stress signaling.^68,71,76^ These molecular changes generally coincide with improved photosynthetic performance and stress tolerance, suggesting that CQDs can influence coordinated regulatory networks rather than isolated biochemical processes. Metabolomic studies similarly report changes in carbohydrate metabolism, amino acid biosynthesis, osmoprotectant accumulation, and antioxidant pathways following CQD treatment,^71,112^ indicating that enhanced photosynthetic energy capture can be coupled to resource allocation and stress adaptation.

Several studies provide particularly clear examples of this integrated response. Titanium-doped CQDs improved salinity tolerance in rice through coordinated transcriptomic and metabolomic changes involving photosynthesis, ion homeostasis, antioxidant defense, and stress signaling.^76^ CQD priming produced a comparable systems-level response in *Elymus sibiricus* under drought by coordinating photosynthesis with carbohydrate metabolism and stress defense,^71^ while red-emissive CQDs altered photosynthesis-related gene networks during growth and delayed senescence in tomato.^68^ Enhanced nutrient acquisition further links these responses to whole-plant resource use, with several CQD systems increasing nutrient uptake alongside chlorophyll content, photosynthetic activity, and biomass.^85,88,113^ Thus, CQD-mediated photosynthetic enhancement appears capable of propagating into carbon–nitrogen metabolism, antioxidant defense, and environmental adaptation rather than remaining confined to the chloroplast.

These observations support a systems-level model in which CQDs influence photosynthesis through the five mechanisms defined above, with downstream effects extending into metabolic and stress-responsive networks.^114–116^ However, the available multi-omics evidence remains predominantly associative. Most studies examine relatively short-term responses in a limited set of crop species, and transcriptomic or metabolomic changes alone do not establish that CQDs directly target the identified pathways. Integrating transcriptomics, metabolomics, proteomics, ionomics, and spatial imaging with targeted pharmacological or genetic intervention will therefore be necessary to distinguish primary CQD responses from downstream physiological consequences. The systems-level framework should consequently be viewed as a supported but still-developing interpretation of CQD action rather than a fully resolved molecular mechanism.

### 5.7 Evidence hierarchy across the proposed mechanisms

The five mechanisms discussed above are supported by different levels of evidence and should therefore not be interpreted as having equivalent mechanistic certainty. Electron transport enhancement represents the strongest evidence tier, because Chandra *et al.* demonstrated an approximately 93% increase in electron-transfer activity using amine-functionalized CQDs in isolated chloroplasts.^65^ Studies in maize, apple, and *Arabidopsis* further support enhanced electron transport following CQD treatment,^66,67,87^ although they do not provide the same degree of mechanistic isolation.

Light harvesting and conversion, redox homeostasis and antioxidant regulation, and chlorophyll biosynthesis and protection occupy an intermediate evidence tier. Spectroscopic, photophysical, and physiological studies support CQD-mediated spectral conversion and excitation-energy transfer.^69,70,94^ More broadly, quantum-dot optical responses are strongly influenced by particle characteristics, surface states, interfacial interactions, and spectral tuning, demonstrating that optical behavior cannot be considered independent of material design.^157,158^ Redox regulation is supported by ROS and antioxidant measurements, stress-response analyses, and defined nanozyme-like activity in iron-doped CQDs.^72–75,89–92^ Similarly, changes in chlorophyll content, chloroplast stability, and photochemical performance support pigment protection, particularly in Mg-, Mg,N-, and Ce-containing systems.^73,74,92^ However, direct interaction of these CQDs with specific photosynthetic or chloroplast targets in intact plants remains insufficiently demonstrated.

Carbon fixation enhancement is classified as indirect evidence. Increases in Rubisco activity, CO_2_ fixation, carbohydrate accumulation, and related metabolic outputs demonstrate that carbon assimilation is affected,^66,106,121,122^ but these responses generally occur alongside changes in light harvesting, electron transport, or whole-plant physiology. They therefore establish a robust functional response without demonstrating a direct interaction between CQD and the Calvin cycle. The evidence also demonstrates that heteroatom doping should not be regarded as a uniform mechanistic strategy. N-doped CQDs provide evidence for an electronically driven response, with maize treatment increasing net photosynthesis by 21.51%, root and shoot carbohydrate contents by 66.43% and 42.03%, respectively, and grain yield by 24.50%.^67^ In contrast, S-containing CQDs are more strongly associated with ROS regulation and protection of the photosynthetic machinery under stress.^89^ Other dopants similarly show distinct functional associations, including Mg with photoprotection,^73,74^ Se with antioxidant regulation,^72,90^ Fe with nanozyme-like ROS scavenging,^75^ and Ce with chlorophyll and redox regulation.^92^ Thus, dopant identity, chemical state, and surface configuration can alter the resulting biological function rather than simply increasing CQD activity.

This interpretation is consistent with the broader quantum-dot literature, which shows that composition, size, surface states, and interfacial structure can substantially modify optical and charge-transfer behavior. For example, Yang *et al.* demonstrated a broadband quantum-dot optical response and tunable spectral behavior in a quantum-dot/polyaniline system, while Xu *et al.* demonstrated tunable aggregation-induced emission behavior and optical waveguiding in nanofiber-based systems.^157,158^ Although these studies are not plant experiments, they provide materials-level evidence supporting the principle that CQD/QD photophysical behavior is strongly dependent on structural and interfacial design rather than being an invariant property of the quantum-dot class. The plant studies discussed above extend this principle to photosynthetic regulation by showing that different CQD compositions can preferentially influence electron transfer, ROS homeostasis, or photoprotection.

The multi-omics evidence discussed in Section 5.6 is complementary rather than an additional mechanism. Transcriptomic and metabolomic studies demonstrate coordinated changes in photosynthesis, carbon metabolism, nutrient acquisition, ion homeostasis, antioxidant defense, and stress signaling,^68,71,76,112^ but remain predominantly associative. Similarly, definitive CQD localization within intact chloroplasts remains insufficient to support higher-level chloroplast targeting. Overall, the hierarchy places electron transport enhancement at the direct level; light harvesting and conversion, redox regulation, and chlorophyll protection at the moderate level; and carbon fixation at the indirect level. Importantly, the available evidence indicates that CQD performance is dopant- and structure-dependent rather than uniformly enhanced by heteroatom incorporation. Future studies that combine matched dopant comparisons, isolated chloroplast assays, spatially resolved imaging, time-resolved spectroscopy, targeted interventions, and multi-omics analyses will be essential for converting these associations into predictive structure-function-performance relationships.

## 6 Agricultural applications of CQDs

The mechanisms established in Section 5 provide the basis for understanding how CQDs influence crop performance across different stages of agricultural production. Their effects extend from seed germination and early seedling establishment to photosynthetic activity, adaptation to abiotic stress, nutrient acquisition, and ultimately biomass and yield. Unlike conventional agricultural inputs that are often designed to target a relatively specific physiological process, CQDs can integrate multiple functions within a single material platform, including modulation of light utilization, redox homeostasis, carbon metabolism, and nutrient-related processes. This multifunctionality arises from the coupling of CQD physicochemical properties with the biological environment rather than from CQD exposure alone. In particular, particle size, surface chemistry, heteroatom composition, optical behavior, and redox properties can determine how CQDs interact with plant tissues and which biological processes are subsequently affected.

Recent studies further demonstrate that CQD performance can be influenced deliberately through precursor selection, heteroatom doping, surface functionalization, and optical or catalytic engineering. Biomass-derived CQDs, heteroatom-doped systems, spectral-converting materials, and nanozyme-like CQDs have therefore been investigated under both favorable and stress conditions, with reported improvements in germination, photosynthetic performance, stress tolerance, nutrient utilization, biomass accumulation, and yield.^117,118^ These observations are consistent with the structure-function-performance relationships discussed in Sections 3–5, but the strength of the causal connection varies among applications. Some studies provide direct mechanistic evidence linking a defined material property to a specific physiological process, whereas others rely primarily on physiological or agronomic endpoints.

**Fig. 4:**
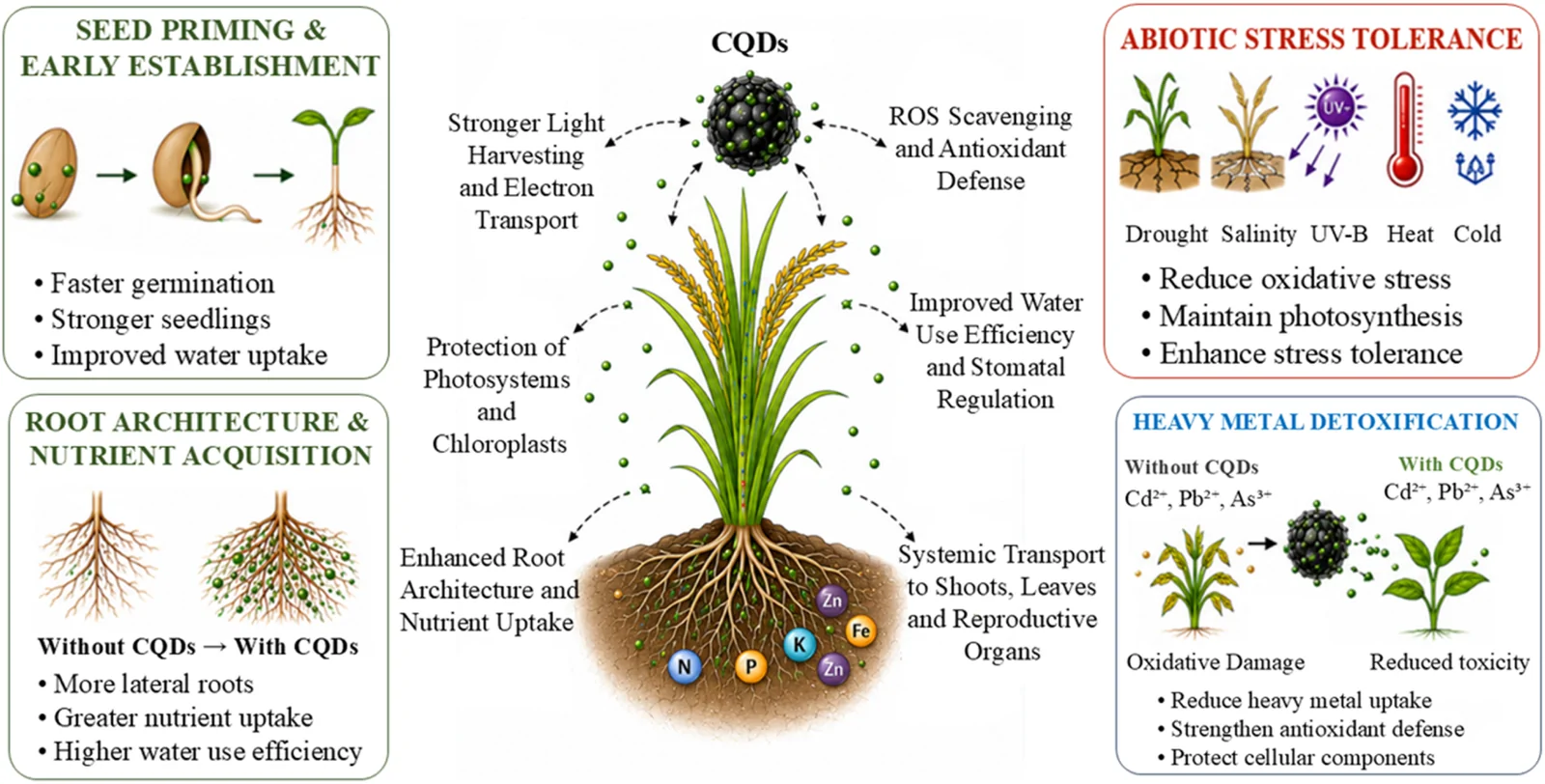
Multifunctional agricultural applications of CQDs, highlighting their roles in seed priming, nutrient acquisition, stress mitigation, heavy-metal detoxification, and sustainable crop productivity through integrated structure-function-performance relationships.

Accordingly, the agricultural applications discussed below are organized by the biological process or technological function targeted. Section 6.1 examines seed priming, where CQD-seed interactions influence germination and early establishment. Section 6.2 considers photosynthetic enhancement, with emphasis on optical and electronic properties, electron transport, carbon fixation, and biomass formation. Section 6.3 addresses abiotic stress mitigation, focusing on redox regulation, ion homeostasis, and preservation of photosynthetic function under adverse conditions. Section 6.4 examines nutrient acquisition and resource-use efficiency, particularly the interactions among root development, nutrient uptake, and carbon–nitrogen metabolism. Finally, Section 6.5 considers advanced delivery systems and smart agriculture, where formulation and system-level engineering determine CQD localization, retention, release, sensing capability, and field applicability. The major agricultural application pathways are summarized in Fig. 4, while representative studies on CQD-mediated seed priming, photosynthetic enhancement, stress resilience, nutrient management, and advanced agricultural technologies are compiled in Table 5.

**Table 5:** Carbon quantum dots for seed priming, stress resilience, and advanced agricultural applications

S. No.	CQD design	Plant system	Application strategy	Primary agricultural function	Key physiological outcome	Quantitative outcome	Technology readiness/agricultural significance	References
1	CDs	Soybean	Foliar application	Nitrogen-use efficiency under drought	Improved nitrogen bioavailability, growth, and seed nutritional quality	Significant increase in seed protein and amino acid content	Links CQD action to nutrient-use efficiency under drought	88
2	Red-emissive CDs	Tomato	Foliar spray	Yield enhancement	Improved photosynthesis and delayed senescence	Plant height ↑10.26%; biomass ↑19.81%	Transcriptomic evidence linking CQDs with yield improvement	68
3	Algae-derived CQD	Basil	Foliar application	Photosynthetic enhancement	Enhanced photosynthesis and electron transport	Photosynthetic rate ↑44.65%; biomass ↑30%	Spectral-converting nano-biostimulant for crop productivity	69
4	CQD	*Elymus sibiricus*	Seed priming	Drought resilience	Improved photosynthesis, antioxidant defense, and carbohydrate metabolism	Significant enhancement in biomass and drought tolerance	Multi-omics validation of drought adaptation	71
5	PAA@Se-CQD	Rapeseed	Nano-priming	Salinity resilience	Improved germination under salt stress	Significant enhancement under salinity	Advanced nano-priming system for saline agriculture	72
6	Mg-CQD	Rice	Foliar application	Salinity mitigation	Enhanced photosynthesis and biomass accumulation	Significant improvement under salt stress	Multifunctional salinity-protection platform	73
7	Fe-CQD nanozyme	Lettuce	Foliar application	Arsenic-stress mitigation	Enhanced cellular protection and stress tolerance	Significant reduction in oxidative stress	First nanozyme-enabled crop-protection platform	75
8	Ti-CDs	Rice	Seed priming	Salinity stress mitigation	Enhanced germination and metabolic reprogramming	Root length ↑90.22%; biomass ↑31.01%	Multi-omics validation of stress tolerance	76
9	Carbon dots	Pakchoi	Hydroponic supplementation	Nutrient-use efficiency	Enhanced nutrient uptake, PSII efficiency, and growth	Dose-dependent growth enhancement	Establishes nutrient uptake-photosynthesis relationship	85
10	N-CQD	Rice	Seed priming	Arsenic stress mitigation	Improved germination, chlorophyll content, and antioxidant defense	Germination ↑14%; chlorophyll ↑59%; as ↓58%	Sustainable rice cultivation in contaminated soils	86
11	Se,N-CQD	Rice	Foliar application	Salt-stress adaptation	Enhanced antioxidant defense and growth	Improved salinity resilience	Integration of nanotechnology and stress adaptation	90
12	CQDs	*Brassica juncea*	Foliar spray	Photosynthetic enhancement	Increased CO_2_ assimilation and Rubisco activity	CO_2_ assimilation ↑58%; Rubisco ↑33%	Mechanistic evidence for CQD-enhanced photosynthesis	106
13	Biomass/waste-derived C-dots	Chickpea, barley, mung bean, wheat	Seed soaking	Germination enhancement	Improved germination across four crop species	Significant enhancement reported	Multi-crop validation using waste-derived precursors	120
14	Put-CQD	*Vitis vinifera* (grapevine)	Foliar priming	Salinity stress mitigation	Improved K^+^ content, photosynthetic pigments, and chlorophyll fluorescence; reduced Na^+^, malondialdehyde (MDA), and H_2_O_2_	Most effective at 10 mg L^−1^	Functionalized-CQD priming platform for viticulture	123
15	Zn-doped biochar-CQD + proline	Chili	Combined soil and foliar application	Drought-stress mitigation	Reduced malondialdehyde content; improved growth and yield	Significant yield improvement under combined treatment	Combines metal-doped biochar-CQDs with osmoprotectant priming	124
16	CQDs	Soybean	Foliar application (2-year field trial)	Drought-stress mitigation	Improved PSII efficiency, chlorophyll content, leaf area, and grain yield	Yield ↑25%; PSII efficiency ↑19%; chlorophyll ↑18%; leaf area ↑21%	First field-scale validation of CQD-mediated drought protection	125
17	CDs	Sweetpotato	Foliar application	Nitrogen-use efficiency	Enhanced nitrogen uptake and assimilation *via* cytokinin-mediated phosphate starvation response protein 1 (CEPD1)-dependent shoot-to-root signaling	Improved field yield reported	First mechanistic link between CQDs and CEPD-mediated nitrogen signaling	126
18	N-doped CDs	Strawberry	Soil/fertilizer application	Growth and yield enhancement	Improved vegetative growth and fruit yield	Significant yield increase reported	Plant-derived CQDs evaluated as a standalone biofertilizer	127
19	Putrescine-CQD	Grapevine	Foliar + soil application	Cadmium-stress mitigation	Reduced oxidative damage and improved chlorophyll fluorescence	Significant Cd-stress alleviation	Functionalized CQDs for heavy-metal management	128
20	GTACD hydrogel-CQD	Maize	Seed coating	Drought resilience	Improved germination and seedling growth	Significant enhancement under drought	Smart seed-coating technology	129
21	GQDs-Cys	Apricot	Foliar spray	Drought stress mitigation	Enhanced antioxidant defense and chlorophyll retention	Chlorophyll *a* increased from 2.76 to 3.58 mg g^−1^ FW	Nano-biostimulant for perennial fruit crops	133
22	Biomass-derived CQDs	Lettuce	Foliar application	Cold-stress tolerance	Improved photosynthetic activity under chilling conditions	Significant improvement under cold stress	Extends CQD application to low-temperature agriculture	134
23	FM-CQD	*Curcuma longa*	Foliar application	Crop productivity enhancement	Increased chlorophyll and curcumin biosynthesis	Significant increase in curcumin content and growth	CQD-mediated enhancement of secondary metabolism	135
24	N-CQD	Maize	Foliar application	Drought adaptation	Improved photosynthesis and biomass accumulation	Significant enhancement under drought	Demonstrates CQD-mediated drought resilience	136
25	Carbon-dot nanoparticles	Sweet potato	Hydroponic supplementation	Salinity stress mitigation	Enhanced photosynthesis and ion homeostasis	Improved Fv/Fm and K^+^/Na^+^ ratio	ROS-regulated salt-tolerance mechanism	137
26	Carbon dots	Cotton	Seed nanopriming	Salinity stress mitigation	Improved germination, ROS signaling, and Na^+^ homeostasis	Root length ↑205.2%; germination ↑32%	Mechanistic evidence for H_2_O_2_-mediated salt tolerance	138

This organization allows the material-to-plant relationship to be followed across progressively broader levels of biological organization: from CQD-seed or CQD-tissue interaction, through specific physiological mechanisms, to whole-plant performance and agricultural deployment. Rather than treating these applications as independent outcomes, the following sections therefore evaluate how differences in CQD design influence biological accessibility and function, how those functions are expressed as measurable physiological responses, and where the available evidence remains insufficient to establish a direct causal relationship with agronomic performance.

### 6.1 Seed priming

Germination and early seedling establishment represent one of the most direct agricultural applications of CQDs because seeds provide an accessible interface through which material properties can influence subsequent plant development. At this stage, CQD performance depends initially on interactions with the seed surface and hydrated tissues, followed by uptake and biological activity. CQDs can interact with seed coats, facilitate water uptake, and stimulate early metabolic processes, providing a plausible material-to-plant pathway for accelerated germination and seedling establishment.^49,96,119^ Across different crops and precursor types, CQD priming has been associated with improved germination, seedling vigor, root development, chlorophyll accumulation, and early biomass production, with reported responses linked to enhanced water imbibition, reserve mobilization, antioxidant regulation, and earlier establishment of photosynthetic activity. Basil-derived CQDs, for example, improved seedling establishment through enhanced physiological activity during germination.^83^ The reproducibility of this response across material sources is further supported by biomass- and plastic-waste-derived CDs, which enhanced germination in chickpea, barley, mung bean, and wheat following simple seed soaking.^120^

The effects of priming can extend beyond the germination window into subsequent plant development. CQD-primed plants have exhibited improved photosynthetic performance, root architecture, and stress tolerance at later developmental stages,^76,86,97^ suggesting that early CQD exposure can induce physiological changes that persist beyond the initial germination response. However, these benefits are strongly dependent on exposure conditions. Low-to-moderate CQD concentrations generally promote germination and seedling development, whereas excessive exposure can diminish the response or disturb cellular homeostasis.^47,107^ Lemon-balm seedlings, for instance, showed maximum germination and antioxidant responses at 100 mg L^−1^ CQDs, whereas both lower and higher concentrations produced weaker responses.^97^ Thus, dose is an important determinant of whether CQD exposure produces a beneficial priming effect or becomes physiologically unfavorable.

The material properties governing this response are not limited to concentration. Particle size and surface chemistry can influence interaction with and access through the seed coat, while surface functional groups can affect hydration, adhesion, and interactions with cellular components. Following uptake, heteroatom composition and associated electronic or redox properties may influence antioxidant regulation and metabolic activation. Selenium-containing CQDs, for example, were associated with improved germination and salt resilience through ROS-related regulation,^72^ while N-doped CQDs improved germination and chlorophyll accumulation while reducing arsenic accumulation in rice.^86^ Ti-containing CQDs similarly enhanced germination, root development, and biomass under salinity through broader metabolic reprogramming.^76^ These examples indicate that priming responses arise from the interaction between CQD accessibility, surface-mediated interactions, and post-uptake biological activity rather than from exposure alone.

The persistence of CQD-mediated effects is nevertheless material-, dose-, crop-, and stress-dependent. Early improvements in germination do not necessarily translate into greater biomass or yield, and evidence connecting molecular or biochemical changes during priming with long-term field productivity remains limited. CQD nano-priming should therefore be viewed as a promising route for establishing favorable early developmental conditions rather than as a universally effective treatment. Future studies should establish standardized dose-response relationships, quantify CQD uptake and retention within seeds, and determine whether early molecular and physiological responses consistently translate into improved crop performance across soil environments and field conditions.

### 6.2 Enhancement of photosynthetic performance and crop productivity

Photosynthetic enhancement provides a direct route through which CQD properties can influence plant productivity. Following uptake and interaction with plant tissues, CQDs can affect light harvesting, photosynthetic electron transport, chlorophyll stability, redox balance, and carbon fixation. These processes determine the efficiency with which absorbed radiation and inorganic carbon are converted into carbohydrates and biomass. Consequently, CQD-mediated increases in photosynthetic performance can extend beyond instantaneous physiological measurements to influence biomass accumulation, crop quality, and yield. The evidence, however, indicates that these responses arise through several distinct material-dependent mechanisms rather than from a universal photosynthetic effect.

Early studies demonstrated a connection between CQD treatment, carbon fixation, and crop productivity. CQD application increased Rubisco activity and grain yield in rice, providing a direct physiological link between enhanced carbon assimilation and productivity.^121^ Comparable improvements in photosynthetic performance and biomass were reported for nitrogen-doped CQDs in maize and CQD-treated wheat.^67,122^ In maize, nitrogen incorporation is particularly relevant because heteroatom doping alters the electronic characteristics of CQDs and can facilitate charge-transfer processes associated with photosynthetic regulation, thereby increasing photosynthesis and carbohydrate accumulation.^67^ These findings suggest that compositional modification can influence a specific photosynthetic function, thereby affecting downstream carbon allocation and plant growth.

CQDs can also enhance photosynthesis through optical rather than primarily biochemical mechanisms. Algae-derived CQDs increased basil photosynthetic efficiency and biomass through UV-to-visible spectral conversion, effectively extending the usable fraction of incident radiation.^69^ Orange-emissive CQDs similarly enhanced chloroplast activity and carbon assimilation in green bean, indicating that emission properties can influence photosynthetic performance when their spectral characteristics are compatible with endogenous pigments.^94^ Coffee-ground-derived CQDs provide a related artificial-antenna mechanism in rice, in which excitation-energy transfer increased photosynthesis and soluble-sugar accumulation, as supported by spectroscopic and computational evidence.^70^ Together, these studies demonstrate that CQD optical properties can serve as active design parameters rather than merely as characterization features, although the magnitude of the response depends on spectral overlap, particle localization, and the plant photosynthetic apparatus's ability to utilize the additional excitation energy.

CQDs may additionally improve photosynthetic performance by protecting the photosynthetic machinery under unfavorable conditions. Biomass-derived CQDs improved photosynthetic activity and cold-stress tolerance in lettuce, suggesting that enhanced photosynthetic function can be maintained when environmental conditions constrain normal photochemistry.^134^ Similarly, FM-CQDs increased chlorophyll accumulation and curcumin biosynthesis in *Curcuma longa*, indicating that CQD-mediated photosynthetic regulation can influence both primary carbon assimilation and secondary metabolism.^135^ Such responses are consistent with a coupled effect in which preservation of chlorophyll and redox balance maintains photosynthetic capacity, while greater carbon availability and reducing power support biomass formation and metabolite biosynthesis.

Electron transport and carbon fixation provide an additional mechanistic link between CQD treatment and productivity. Biomass-derived CQDs enhanced photosynthetic activity and carbon fixation in *Arabidopsis*, whereas biochar-derived CQDs increased CO_2_ assimilation by 58% and Rubisco activity by 33% in green mustard.^66,106^ Nitrogen-doped CQDs likewise increased photosynthesis and carbohydrate accumulation in maize.^67^ These observations connect CQD-mediated regulation of photosynthetic processes with measurable carbon-assimilation responses. Nevertheless, the causal sequence should be interpreted cautiously. Although Rubisco activity and CO_2_ assimilation are direct physiological indicators, most studies do not experimentally separate CQD effects on carbon fixation from upstream changes in light harvesting, electron transport, chlorophyll stability, or redox regulation.

The consequences of enhanced photosynthetic competence may also accumulate over longer developmental periods. Red-emissive CDs delayed senescence and increased tomato biomass while modulating photosynthesis-related gene networks,^68^ suggesting that maintaining functional photosynthetic tissues for longer periods could increase cumulative carbon assimilation. Collectively, the available evidence indicates that CQDs can influence photosynthesis through at least three interacting routes: spectral modification of incident light, regulation of photosynthetic electron-transfer processes, and preservation of chlorophyll and redox homeostasis. These mechanisms may converge on increased carbon assimilation and biomass production, but their relative importance depends on particle size, surface chemistry, heteroatom composition, optical properties, dose, plant species, and application route.^47,107^

Overall, CQD-mediated photosynthetic enhancement is best understood as a material-specific process rather than a generic consequence of nanoparticle exposure. The strongest evidence links defined CQD properties to measurable changes in light utilization, electron transport, chlorophyll status, Rubisco activity, or CO_2_ assimilation, whereas evidence connecting these mechanisms directly to field-scale yield remains more limited. Establishing such links will require simultaneous characterization of CQD optical and electronic properties, plant uptake and localization, photosynthetic parameters, carbon allocation, and final agronomic performance across realistic environmental conditions.

### 6.3 Abiotic stress mitigation

Drought, salinity, heavy-metal contamination, temperature extremes, and ultraviolet radiation constrain crop productivity through interconnected disturbances in water relations, ion homeostasis, redox balance, chlorophyll stability, and photosynthetic electron transport. CQDs can alleviate these effects by modifying stress-responsive processes through their surface chemistry, heteroatom composition, redox activity, and, in some cases, optical properties. The resulting protection is therefore highly dependent on the type of stress and the material used, rather than representing a uniform response to CQD exposure.

Salinity is the most extensively investigated stress condition. Excess Na^+^ and Cl^−^ disrupt water relations and ion homeostasis, increase ROS formation, and impair photosynthetic function. Magnesium-containing CQDs improved photosynthetic performance and chloroplast stability under salinity, whereas selenium-containing CQDs enhanced antioxidant defense and stress signaling.^72,73,90^ Surface functionalization provides an additional route for tailoring this response. In *Vitis vinifera* cv. ‘Sultana’, Gohari *et al.* reported that Put-CQD priming, particularly at 10 mg L^−1^, improved K^+^ retention, photosynthetic pigments, chlorophyll fluorescence, and Soil Plant Analysis Development (SPAD) values while reducing Na^+^ accumulation, electrolyte leakage, malondialdehyde (MDA), and H_2_O_2_ under salinity.^123^ In the same cultivar, Panahirad *et al.* investigated Put-CQD treatment under cadmium stress and found that foliar application at 25 and 50 mg L^−1^ reduced Cd-induced losses in leaf fresh and dry weight and improved photosynthetic pigment and chlorophyll–fluorescence parameters, with the stronger response at 25 mg L^−1^.^128^ These studies represent independent examples of stress-specific Put-CQD protection and should not be interpreted as evidence for a single treatment being simultaneously validated against salinity and cadmium. Other CQD compositions provide more integrated regulation of ionic and oxidative stress. Se,N-co-doped CQDs combine antioxidant effects with improved ion homeostasis,^91^ while Ti-doped CQDs improved salinity tolerance in rice through coordinated transcriptomic and metabolomic changes involving photosynthesis, ion regulation, antioxidant defense, and stress signaling.^76^ Carbon-dot nanoparticles similarly improved salt tolerance in sweet potato by maintaining photosynthetic efficiency, ion homeostasis, and ROS balance.^137^ In cotton, mechanistic evidence indicates that carbon-dot seed nanopriming activates NADPH oxidase-dependent H_2_O_2_ signaling and Ca^2+^-associated SOS regulation, enhancing SOS1/NHX7-mediated Na^+^ homeostasis. This response increased germination by 32% and root length by 205.2%, while reducing Na^+^ accumulation by 15.3%.^138^ Together, these studies indicate that effective salinity protection can involve coordinated regulation of ROS signaling, Ca^2+^-dependent stress responses, ion transport, and photosynthetic function.

Drought presents a related but mechanistically distinct challenge, primarily through reduced water availability, stomatal conductance, and carbon assimilation, accompanied by increased oxidative stress. CQD priming improved drought resilience in *Elymus sibiricus* through coordinated regulation of photosynthesis, carbohydrate metabolism, and stress defense.^71^ Nitrogen-doped CQDs similarly enhanced carbon assimilation and photosynthetic efficiency in drought-stressed systems.^62,67^ Graphene quantum dots (GQDs-Cys) improved drought tolerance in apricot by enhancing chlorophyll retention and antioxidant defense, demonstrating the relevance of surface functionalization to perennial fruit crops.^133^ N-doped CQDs further improved photosynthesis, biomass accumulation, and drought resilience in maize.^136^ Collectively, these responses suggest that drought protection depends on maintaining photosynthetic capacity, limiting oxidative damage, and sustaining carbon metabolism, rather than on a single protective pathway. CQD design can also be adapted to oxidative and radiation stresses. Sulfur-containing CQDs exhibit ROS-scavenging activity that can help preserve photosynthetic machinery under stress,^89^ whereas Mg,N-co-doped CQDs improved photosynthetic stability under UV-B exposure through combined photoprotection and antioxidant regulation.^74^ These examples illustrate the importance of matching material chemistry to the dominant stress mechanism. A CQD optimized for ROS regulation, for example, may have a different functional advantage from one designed primarily for ion homeostasis or optical photoprotection.

Heavy-metal stress introduces additional constraints through chlorophyll degradation, ROS accumulation, ion imbalance, and disruption of nutrient metabolism. N-doped CQDs improved arsenic tolerance in rice while preserving photosynthetic activity,^86^ whereas cerium-containing CQDs supported chlorophyll biosynthesis and redox regulation under comparable stress conditions.^92^ Iron-doped nanozyme-like CQDs provide a distinct approach through enzyme-mimetic ROS-scavenging activity,^75^ potentially supporting redox protection during prolonged oxidative stress. However, the proposed persistence advantage of nanozyme-like CQDs remains a design hypothesis rather than an established agricultural benefit and requires validation under realistic soil and field conditions. CQDs may also complement rather than replace conventional stress-management inputs. Zinc-doped biochar-CQDs combined with the osmoprotectant proline reduced MDA accumulation and improved growth and yield in chili under drought stress.^124^ This finding suggests that CQD functionality can be integrated with established agricultural strategies, with their redox, electronic, or nutrient-related properties potentially complementing osmoprotectants and other stress-management inputs.

**Fig. 5:**
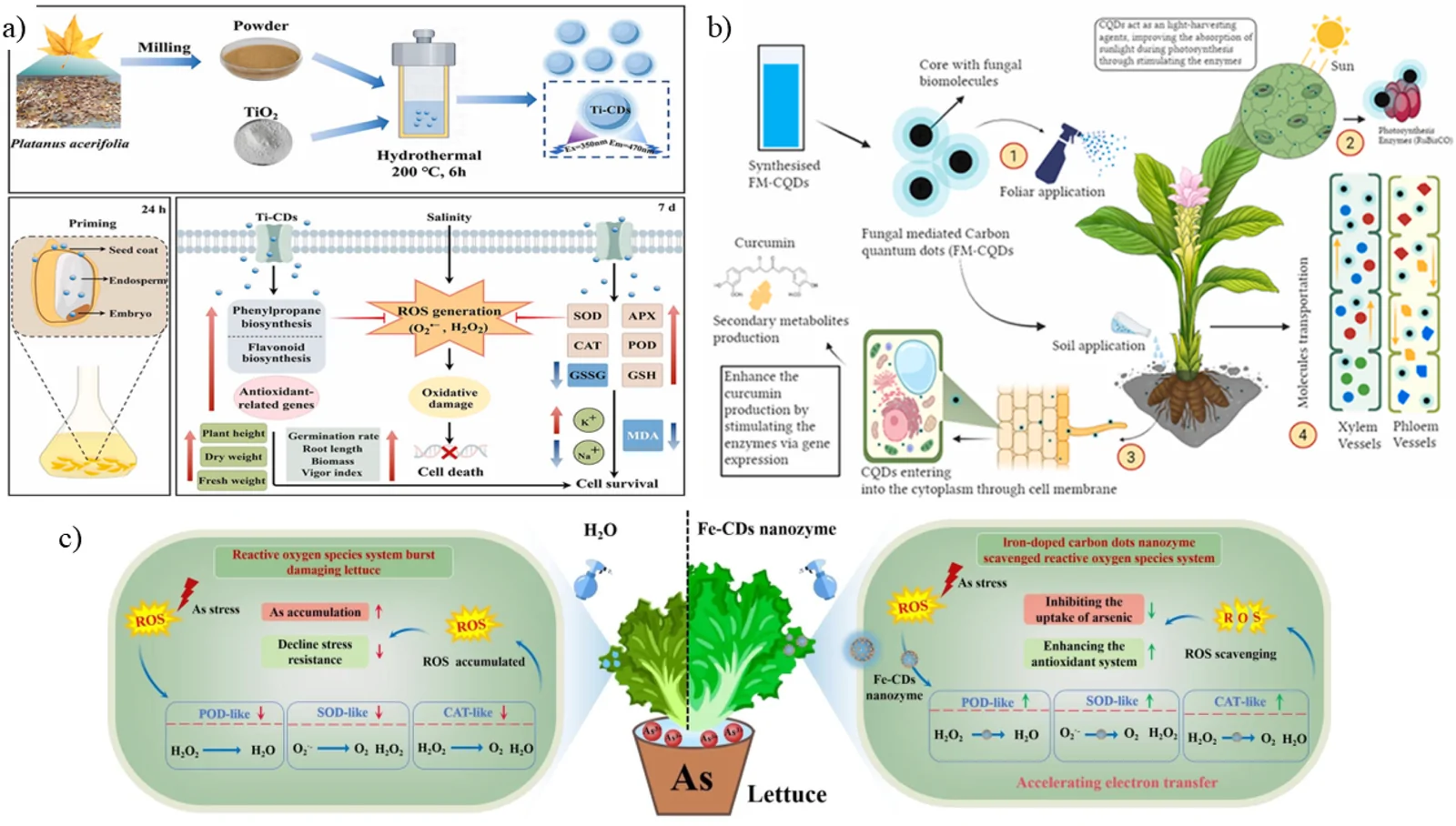
Applications of CQDs in sustainable agriculture. (a) Ti-doped carbon dots (Ti-CDs) enhance seed germination and salinity tolerance by regulating ROS and antioxidant defense. (b) Fungal-mediated CQDs (FM-CQDs) improve photosynthesis, antioxidant activity, and metabolite production following foliar or soil application. (c) Fe-CD nanozymes alleviate arsenic-induced oxidative stress in lettuce through ROS scavenging and enzyme-mimetic antioxidant activity. Reproduced with permission from Elsevier: (a) *Food Bioscience*, License No. 6300610046532; (b) *Pesticide Biochemistry and Physiology*, License No. 6300610677455; (c) *Chemical Engineering Journal*, License No. 6300610498549.

Importantly, the evidence base is beginning to extend beyond controlled greenhouse and hydroponic experiments. A two-year field trial in soybean reported that foliar CQD application increased PSII efficiency by 19%, chlorophyll content by 18%, and leaf area by 21% under field drought conditions, resulting in a 25% increase in grain yield across two growing seasons.^125^ This study provides stronger evidence that CQD-mediated preservation of photosynthetic function can persist under variable field conditions. Nevertheless, broader validation across soil types, climates, crop genotypes, application regimes, and multiple seasons remains necessary. Representative examples of engineered CQDs for sustainable agricultural applications are presented in Fig. 5. Overall, mitigation of abiotic stress demonstrates that CQD efficacy depends on matching material functionality to the dominant physiological constraint. Surface chemistry and heteroatom composition can influence redox regulation and signaling, while specific CQD formulations can additionally affect ion homeostasis, chlorophyll stability, or photoprotection. The most compelling evidence, therefore, comes from studies that connect a defined material property to a measurable stress-response mechanism and, subsequently, to plant performance. Future work should move beyond reporting increases in stress tolerance alone and establish quantitative relationships among CQD dose, uptake, ROS, and ion signaling, photosynthetic preservation, and agronomic outcomes under field-relevant conditions.

### 6.4 Nutrient acquisition and resource-use efficiency

Nutrient acquisition is closely linked to photosynthetic productivity because nitrogen, phosphorus, and micronutrients support chlorophyll biosynthesis, photosynthetic enzymes, electron transport, carbon assimilation, and biomass formation. CQDs may influence this process indirectly through changes in root development, nutrient availability, and plant signaling, or more directly through interactions between their surface functionalities and nutrient ions. Thus, the relevance of CQDs to resource-use efficiency extends beyond their potential role as nutrient carriers to encompass their ability to modulate the biological processes governing nutrient acquisition and utilization.

Several studies have reported enhanced uptake of nitrogen, phosphorus, and micronutrients following CQD treatment, generally accompanied by increases in chlorophyll content, photosynthetic activity, and biomass.^85,88,113^ CQD application in coriander improved nutrient acquisition together with photosynthetic performance,^113^ while a comparable association between nutrient uptake and photosynthetic capacity was observed in pakchoi.^85^ These responses are potentially reciprocal: improved nutrient availability can support pigment formation, photosynthetic enzymes, and metabolic activity, whereas increased carbon assimilation can provide the energy and carbon skeletons required for nutrient assimilation. However, such associations do not by themselves establish whether CQDs directly regulate nutrient transport or whether improved nutrient status is a downstream consequence of enhanced plant growth and metabolism.

Root architecture provides a more direct interface between CQD exposure and belowground resource acquisition. Ce,N-co-doped CQDs promoted root development and nutrient acquisition through coordinated regulation of redox homeostasis and growth-associated signaling.^93^ A more specific molecular mechanism was demonstrated in sweetpotato, where foliar-applied CDs enhanced nitrogen uptake and assimilation through cytokinin-mediated phosphate starvation response protein 1 (CEPD1)-dependent shoot-to-root signaling.^126^ This evidence is particularly important because it identifies a well-defined long-distance signaling pathway linking aboveground CQD application to belowground nitrogen acquisition, rather than relying solely on increased root biomass as an indicator of improved nutrient uptake. It also demonstrates that the effects of CQDs can propagate between plant organs through endogenous signaling networks.

Carbon–nitrogen interactions provide an additional link between resource acquisition and plant productivity. CDs improved nitrogen bioavailability in soybean under drought, supporting plant growth and seed nutritional quality,^88^ while CQD treatment also enhanced amino-acid biosynthesis and nitrogen assimilation under drought.^112^ These findings indicate coordinated regulation of carbon and nitrogen metabolism, in which photosynthetic carbon supply can support nitrogen assimilation while adequate nitrogen availability sustains chlorophyll formation and photosynthetic activity. The relationship is therefore potentially reciprocal rather than a simple sequence in which CQDs increase nutrient uptake and nutrients subsequently improve photosynthesis.

The mechanisms underlying improved nutrient-use efficiency nevertheless remain incompletely resolved. Enhanced carbon assimilation may increase the energy available for nutrient uptake and assimilation, while improved redox regulation can preserve membrane integrity and transporter activity under stress. At the same time, CQD surface functional groups may interact with nutrient ions, altering their availability or transport, and heteroatom doping may modify root activity, redox signaling, and metabolic responses. Distinguishing these direct and indirect contributions is important because increased tissue nutrient concentrations do not necessarily demonstrate direct regulation of nutrient transport by CQDs. Evidence is also beginning to extend beyond controlled hydroponic and pot experiments. Plant-derived N-doped CDs applied as a standalone soil amendment improved vegetative growth and fruit yield in strawberry, demonstrating that CQDs can function as biofertilizer-like inputs rather than solely as supplementary biostimulants.^127^ This represents an important translational step because the material was evaluated using conventional agronomic endpoints, including growth and yield, rather than only biochemical or physiological indicators. Overall, CQD-mediated improvements in nutrient-use efficiency appear to arise from interactions among root development, nutrient acquisition, endogenous signaling, and carbon–nitrogen metabolism. The available evidence is strongest where CQD treatment has been linked to a defined signaling pathway or to coordinated changes in nutrient uptake and plant metabolism, whereas direct regulation of nutrient transport remains less firmly established. Future studies should therefore combine nutrient-flux measurements, transporter-level analyses, isotope tracing, root imaging, and multi-season field trials to distinguish direct nutrient-regulatory effects from downstream consequences of enhanced photosynthesis, growth, or stress tolerance.

### 6.5 Advanced agricultural delivery systems and smart agriculture

The translation of CQDs from laboratory demonstrations to practical agricultural use requires control over where, when, and for how long their biological functions are expressed. Free CQDs can undergo dilution, aggregation, transformation, or removal from the application site, making their effective exposure difficult to predict. Engineered delivery systems, therefore, provide an additional level of control beyond CQD composition itself. Hydrogels, polymeric carriers, nanocomposites, nanozyme formulations, and emerging sensing platforms can regulate CQD retention, localization, release, and stability while enabling their integration with established agricultural practices.^143^ In this context, agricultural performance depends not only on the intrinsic properties of the CQD but also on the behavior of the complete CQD-carrier-plant system.

Controlled-release formulations are particularly relevant for applications in which prolonged or localized exposure is desirable. Incorporation of CQDs into hydrogels, polymeric matrices, nanocomposites, or biodegradable carriers can reduce material loss and modify release kinetics at seeds, roots, or foliage. Carbon-dot-embedded hydrogels, for example, promoted maize germination and growth under drought stress, suggesting that a water-retaining carrier can simultaneously improve water availability and maintain CQD activity near the target tissue.^129^ Such formulations may therefore provide advantages over free-particle application by controlling both the physical environment and CQD exposure. Seed coatings and rhizosphere-targeted formulations are especially attractive because localized retention could reduce off-target dispersion and the need for repeated applications. CQD catalytic functions can also be incorporated into delivery systems. Iron-doped CQDs exhibit enzyme-mimetic ROS-scavenging activity, making them potential candidates for formulations that provide localized redox protection.^75^ However, the practical value of this approach depends on whether catalytic activity can be retained after incorporation into a carrier and maintained under the chemical and biological conditions encountered in soil or plant tissues. Factors such as pH, ionic strength, organic matter, adsorption, and interactions with biomolecules may substantially alter nanozyme activity. Therefore, enhanced catalytic performance measured under controlled laboratory conditions should not automatically be interpreted as sustained activity in agricultural environments.

The optical and surface properties of CQDs also enable integration with agricultural sensing and precision management systems. CQD fluorescence and environmentally responsive surface chemistry have been explored for monitoring plant physiological status, nutrients, stress-associated signals, and agricultural contaminants.^130,144^ This introduces a potentially important dual-function concept in which a CQD platform can both perform a biological function and provide information about plant or environmental conditions. Integration with sensor networks and responsive agricultural technologies could enable treatment decisions based on measured crop status rather than fixed application schedules.^131^ Nevertheless, most current demonstrations remain at the proof-of-concept or controlled-environment stage, and reliable integration of CQD-based sensing with farm-scale data systems and automated delivery remains largely unvalidated. A further development is the design of multifunctional formulations that combine several agronomic functions within a single platform. Such systems may integrate photosynthetic regulation, stress protection, nutrient management, and sensing, reflecting the interconnected nature of the processes discussed in Sections 6.1–6.4.^143^ Improved nutrient acquisition can support chlorophyll biosynthesis and photosynthesis; enhanced photosynthetic carbon supply can contribute to stress adaptation; and redox regulation can protect both photosynthetic and nutrient-transport processes. A delivery system that maintains CQD activity at an appropriate tissue concentration and time scale could therefore coordinate several responses rather than relying on repeated application of independent inputs.

However, increasing functional complexity also increases the requirements for material optimization and quality control. Particle size, surface charge, heteroatom composition, functionalization, polymer conjugation, carrier composition, stability, release kinetics, and biological dose may all interact to determine final performance. Biomass-derived and environmentally benign CQDs offer promising starting materials for safe-by-design agricultural formulations,^61,64,72^ but green synthesis alone does not ensure environmental safety, reproducibility, or manufacturing consistency. Variability in biomass precursors can alter CQD composition and surface chemistry, thereby affecting both delivery behavior and biological activity. Standardized synthesis and physicochemical characterization will therefore be essential for translating laboratory-scale formulations into reproducible agricultural products.

From an application perspective, the central advance of engineered delivery systems is therefore the ability to control the CQD-plant interface rather than simply increases the number of functions incorporated into the material. CQD composition determines intrinsic optical, electronic, and redox properties, whereas formulation architecture determines exposure, localization, retention, and release. Their interaction ultimately determines whether a desired biological response can be reproduced under realistic agricultural conditions. This distinction is particularly important because the CQD that performs best in a controlled laboratory assay may not be the formulation that performs best under variable soil chemistry, rainfall, temperature, microbial activity, and crop-management practices. Most delivery and smart-agriculture approaches nevertheless remain at laboratory or greenhouse scale. Field validation should establish whether controlled release improves CQD efficacy under variable environmental conditions, whether formulation components alter environmental fate or persistence, and whether the additional processing required for engineered systems is economically justified. Long-term studies should also determine how repeated application affects soil microbial communities, CQD transformation, plant uptake, and potential accumulation in edible tissues. Thus, the principal promise of advanced CQD platforms lies not simply in combining multiple functions, but in achieving spatially and temporally controlled activity that can connect material design with reproducible plant responses and ultimately with practical agricultural performance.

## 7 Challenges, biosafety considerations, and future perspectives

The mechanistic and agronomic case for CQDs assembled in Sections 3 through 6 is, by this point, substantial. It is also almost entirely a laboratory-and-greenhouse case. Translating it into field-deployable agricultural technology requires confronting three distinct categories of unresolved problems: mechanistic, biosafety, and translational. Each is examined in turn below.

### 7.1 Mechanistic and technical challenges

Despite substantial evidence that CQDs enhance photosynthesis and plant productivity, the molecular basis of these effects remains incompletely resolved. Most studies report changes in photosynthesis, chlorophyll, biomass, or stress tolerance without experimentally distinguishing among light harvesting, electron transport, redox regulation, chlorophyll protection, and carbon fixation. Direct evidence is strongest for CQD-mediated electron transport, particularly from chloroplast-level studies,^65,66^ whereas several other mechanisms remain supported mainly by physiological, biochemical, photophysical, transcriptomic, or metabolomic evidence.

CQD activity is also strongly influenced by uptake and intracellular localization. Particle size, surface charge, and functional groups affect plant interactions and transport, but unambiguous localization within intact chloroplasts has not been demonstrated. Thus, photosynthetic activity depends on both CQD accessibility and the optical, electronic, or redox functionality expressed after uptake.

Heteroatom doping further demonstrates that CQDs should not be considered a mechanistically uniform class. Nitrogen doping primarily illustrates an electronically driven response. In maize, N-doped CQDs increased net photosynthesis by 21.51%, root and shoot carbohydrate contents by 66.43% and 42.03%, respectively, and grain yield by 24.50%, consistent with enhanced photoelectron-transfer processes.^67^ In contrast, S-containing CQDs are more strongly associated with ROS regulation and protection of the photosynthetic machinery under stress.^89^ Thus, N- and S-containing CQDs can produce beneficial plant responses through different functional pathways. Other dopants show similarly distinct behavior: Mg-containing CQDs are associated with chlorophyll protection and photoprotection,^73,74^ Se-containing CQDs with antioxidant and stress signaling,^72,90^ Fe-containing CQDs with nanozyme-like ROS scavenging,^75^ and Ce-containing CQDs with chlorophyll maintenance and redox regulation.^92^

Importantly, direct head-to-head comparisons of differently doped CQDs remain limited. Differences in particle size, surface chemistry, dopant loading, dose, and synthesis conditions can contribute to apparent dopant-specific effects. Standardized characterization and matched-material studies are therefore required to distinguish the contribution of dopant identity from other physicochemical variables.^150,153^ Future studies should combine controlled dopant comparisons with chloroplast assays, intracellular imaging, time-resolved spectroscopy, ROS analysis, biochemical intervention, and multi-omics approaches. Such studies would establish more reliable relationships between dopant chemistry, CQD physicochemical properties, plant accessibility, molecular targets, photosynthetic mechanisms, and agronomic outcomes, providing a stronger basis for rational CQD design.^154–156^

### 7.2 Biosafety, dose-dependent toxicity, and field-scale limitations

The agricultural promise of CQDs is supported by extensive laboratory and greenhouse evidence, but their transition to field deployment remains constrained by long-term safety, environmental fate, scalability, economic viability, and field reproducibility. These considerations are closely interconnected because particle size, surface chemistry, heteroatom composition, dose, and formulation influence not only plant uptake and biological activity but also persistence, mobility, transformation, and interactions with soil and non-target organisms. Recent reviews similarly identify toxicity thresholds, field scalability, environmental fate, and regulatory gaps as major barriers to agricultural translation.^150,153^

A major biosafety concern is the limited understanding of long-term accumulation and transformation in plant-soil systems. CQDs applied to seeds, foliage, or soil may be retained at the application site, internalized and translocated, transformed within plant tissues, adsorbed to soil components, or transferred to non-target organisms. Importantly, ^13^C-tracing demonstrated that foliar-applied CDs accumulated throughout maize tissues and became associated with metabolites including glucose, sucrose, citric acid, glyoxylate, and chlorogenic acid. ^13^C signals were also detected in soil fauna, suggesting potential transfer through the soil food chain, although no pronounced overall change in soil properties was observed during the experimental period.^151^ These findings emphasize that environmental assessment should consider transformation and biological incorporation, rather than evaluating only the persistence of the parent CQD. Environmental behavior is also expected to vary with soil and climatic conditions. Soil pH, ionic strength, mineral composition, organic matter, moisture, temperature, and microbial activity can influence CQD aggregation, adsorption, mobility, and bioavailability. Similarly, rainfall, irrigation, and solar exposure may alter CQD retention and transport following foliar or soil application. Therefore, results obtained under hydroponic or single-soil conditions cannot be assumed to represent behavior across agricultural environments. Future studies should examine CQD transformation and distribution in soil, rhizosphere, plant tissues, drainage water, and relevant non-target organisms across contrasting soil types and climatic regimes.^150,153^

Long-term field validation remains a major translational gap. The two-year soybean field study discussed in Section 6.3 (ref. 125) provides important evidence that CQD-mediated photosynthetic protection can persist under field conditions, but a single crop and location cannot establish broad agricultural reliability. Multi-season and multi-location trials should therefore evaluate different crop genotypes, soil classes, irrigation conditions, climatic regimes, and application strategies. Importantly, such studies should measure not only yield but also photosynthetic performance, nutrient-use efficiency, CQD residues and transformation, soil properties, crop quality, and non-target effects. This would allow the structure-function-performance relationships established under controlled conditions to be tested under realistic environmental variability.

Scalability is another critical constraint. Laboratory CQD synthesis commonly involves small reaction volumes, controlled precursors, extensive purification, and narrowly defined physicochemical properties. Biomass-derived CQDs offer an attractive route because renewable, low-cost feedstocks can reduce precursor costs; however, variability in biomass composition can lead to batch-to-batch differences in particle size, surface chemistry, heteroatom content, and optical properties. Cheng *et al.* demonstrated the agricultural functionality of biomass-derived CDs using a low-cost preparation method, thereby supporting the feasibility of large-scale production.^152^ Nevertheless, scale-up studies should establish synthesis yield, energy and water requirements, purification burden, batch reproducibility, storage stability, and formulation performance. These factors are recognized as important challenges for practical CQD deployment.^150,153^

Economic viability must be evaluated together with scalability. High laboratory efficacy does not necessarily translate into an economically viable agricultural product if the costs of synthesis, purification, formulation, transport, or application are excessive. Future techno-economic assessments should therefore compare the complete cost of CQD production and application with measurable benefits such as increased yield, improved nutrient-use efficiency, reduced application frequency, or enhanced stress tolerance. The relevant benchmark should be the cost per unit agronomic benefit, rather than the material production cost alone.

Finally, regulatory assessment should integrate efficacy with environmental and food-chain considerations. CQDs containing additional elements such as Se, Fe, Ce, or Mg require particular attention because dopant behavior may differ from that of the carbon framework. Long-term accumulation in edible tissues, transformation of dopants, effects on soil microorganisms and invertebrates, and movement into aquatic compartments remain insufficiently characterized for many formulations. Consequently, “green” synthesis or a biomass precursor should not be considered sufficient evidence of environmental safety. Recent agricultural assessments emphasize the need to evaluate toxicity, food-chain exposure, soil interactions, and ecological effects alongside agronomic efficacy. Overall, successful agricultural translation requires an integrated lifecycle framework encompassing CQD design, scalable synthesis, formulation, plant and soil exposure, mechanistic efficacy, long-term field performance, environmental fate, economic viability, and regulatory suitability. The key challenge is therefore not simply demonstrating that CQDs improve plant performance, but establishing whether a reproducibly manufactured formulation can maintain its structure-function-performance relationship across diverse soil-climate systems while providing an economically and environmentally acceptable benefit over multiple seasons. This shift from short-term efficacy toward long-term, field-based, and lifecycle assessment is essential for moving CQDs from promising laboratory materials to credible agricultural technologies.

### 7.3 Future perspectives

Establishing predictive structure-function-performance relationships between CQD physicochemical properties and biological performance should be the field's first priority, since nearly every other goal in this review depends on it. Advanced heteroatom engineering, multifunctional co-doping, and nanozyme-integrated CQDs already offer plausible routes to combining photosynthetic enhancement, stress tolerance, and nutrient-use efficiency in a single material. What is missing is a predictive model that would let researchers design toward a target outcome rather than discover it empirically. Integrating transcriptomics, metabolomics, proteomics, and advanced imaging is the most direct route to that model. Field-scale validation, formulation development, and regulatory standardization deserve comparable priority beyond mechanistic research, not as downstream concerns to address once the science is settled, but as parallel work that should begin now. Controlled-release systems, smart sensors, and precision agriculture integration could plausibly deliver multifunctional platforms that increase productivity while reducing resource use. Biomass-derived CQDs produced from agricultural waste offer a route to environmentally sustainable, economically viable production that is already partly demonstrated, not purely aspirational.^141^ CQDs have earned their position as a versatile class of photosynthetic nano-regulators capable of improving crop performance through several complementary mechanisms. Whether that position translates into practical agricultural technology now depends less on further proof-of-concept studies, of which this review has surveyed a great many, and more on standardized methodology, rigorous biosafety evaluation, and field validation at a meaningful scale. These are solvable problems. They are not yet solved, and treating them as such would be premature.

## 8 Conclusions

Over roughly a decade, CQDs have moved from a curiosity in fluorescence and bioimaging to a credible platform for sustainable agriculture interventions. The evidence assembled in this review supports three conclusions: substantial, well-documented scientific advances; specific, identifiable knowledge gaps rather than a generic call for more research; and a small set of priorities capable of closing most of those gaps if pursued deliberately. The review's principal contribution is a structural account that links CQD design directly to photosynthetic outcomes. Precursor selection, particle size, surface chemistry, and heteroatom doping jointly determine uptake, transport, and chloroplast accessibility, with nitrogen doping shown to simultaneously shape both transport and electron-transfer capacity. Layered onto this is an explicit evidence hierarchy across the five proposed mechanisms: electron-transport facilitation rests on direct, chloroplast-level intervention and is the most strongly supported; carbon assimilation is supported by direct physiological readouts, though usually without isolating it from upstream electron supply; and light harvesting, ROS homeostasis, and the systems-level omics account rest more on correlation than on demonstrated causation. These mechanistic advances have translated into agronomic outcomes across seed priming, photosynthetic enhancement, abiotic stress mitigation, nutrient use efficiency, and emerging delivery and sensing platforms. The clearest sign that this progress extends beyond the laboratory is a two-year field-grown soybean trial in which foliar CQD application increased grain yield by 25% under drought conditions, demonstrating that greenhouse-characterized mechanisms can survive the transition to open agriculture.

Three unequal gaps limit how far this progress can be trusted to generalize. The narrowest is mechanistic: chloroplast-level imaging that would confirm CQD localization is still missing, and four of the five proposed mechanisms remain correlational. Standardization is conceptually simple but remains unresolved in practice, since inconsistent protocols for synthesis and characterization make it difficult to distinguish variable biology from variable materials. The widest gap concerns biosafety and translational readiness: toxicity findings are internally inconsistent, dose-response data favor benefit over harm, chronic and non-target-organism exposure data remain sparse, and no regulatory pathway specific to agricultural nanomaterials yet exists.

The following four priorities are in order of decreasing urgency. Establishing predictive structure-function-performance relationships through integrated omics and imaging should come first; standardized synthesis and reporting protocols should follow closely; dose-response and biosafety research designed to characterize harm as well as benefit is the most urgent relative to the attention it has so far received; and field-scale validation across a genuine range of crops, climates, and soils should proceed in parallel rather than afterward. None of this requires a conceptual breakthrough. CQDs have earned their position as a genuinely versatile class of photosynthetic nano-regulators. Whether that promise translates into deployed technology now depends less on discovering new mechanisms than on standardizing what is already known, rigorously testing it for harm as well as benefit, and validating it under the field conditions it will ultimately need to withstand.

## Conflicts of interest

There are no conflicts to declare.

## Data Availability

No new data were generated or analyzed in this study. All information supporting the findings of this review is included within the manuscript and its cited references.
